# Thrombospondin 1 aggravates cardiac remodeling in heart failure with preserved ejection fraction by inhibiting mitophagy

**DOI:** 10.1016/j.isci.2026.114639

**Published:** 2026-01-07

**Authors:** Xingpeng Bu, Shuo Sha, Zhenzhen Zhang, Sicheng Bian, Shuhui Feng, Chunxia Li, Lei Wang, Huanzhen Chen

**Affiliations:** 1 First Clinical Medical College, Shanxi Medical University, Taiyuan, China; 2 Department of Geriatric Medicine, Shanxi Bethune Hospital, Shanxi Academy of Medical Sciences, Third Hospital of Shanxi Medical University, Tongji Shanxi Hospital, Taiyuan, China; 3 Department of Medicine, The MetroHealth System, Case Western Reserve University, Cleveland, Ohio, USA; 4 Department of Cardiology, First Hospital of Shanxi Medical University, Taiyuan, China

**Keywords:** Cell biology, Omics, Model organism

## Abstract

Heart failure with preserved ejection fraction (HFpEF) accounts for over half of all heart failure cases, but its underlying mechanisms remain unclear. Mitochondrial dysfunction and defective mitophagy are increasingly recognized as central features of HFpEF. Thrombospondin 1 (Thbs1), a matricellular protein involved in cardiovascular remodeling, has not been explored in this context. Here, we show that *Thbs1* expression is elevated in HFpEF myocardium and that *Thbs1* aggravates cardiac dysfunction by inhibiting mitophagy. In a “two-hit” HFpEF mouse model induced by high-fat diet and L-NAME, AAV9-mediated *Thbs1* knockdown improved diastolic function, reduced fibrosis and inflammation, and mitigated PI3K/Akt/mTOR pathway activation revealed by transcriptomic and proteomic profiling. Mechanistically, *Thbs1* silencing restored autophagic flux, enhanced mitochondrial clearance, and preserved mitochondrial homeostasis in cardiomyocytes. These findings identify *Thbs1* as a key suppressor of mitophagy in HFpEF and a potential therapeutic target for this prevalent condition.

## Highlights

• *Thbs1* is upregulated in myocardium of HFpEF mice and correlates with remodeling
• *Thbs1* knockdown alleviates fibrosis, hypertrophy, and oxidative stress in HFpEF mice
• *Thbs1* suppresses mitophagy by activating the PI3K/Akt/mTOR signaling pathway
• Targeting *Thbs1* restores mitochondrial homeostasis and improves diastolic function in HFpEF

## Graphical abstract

**Figure:**
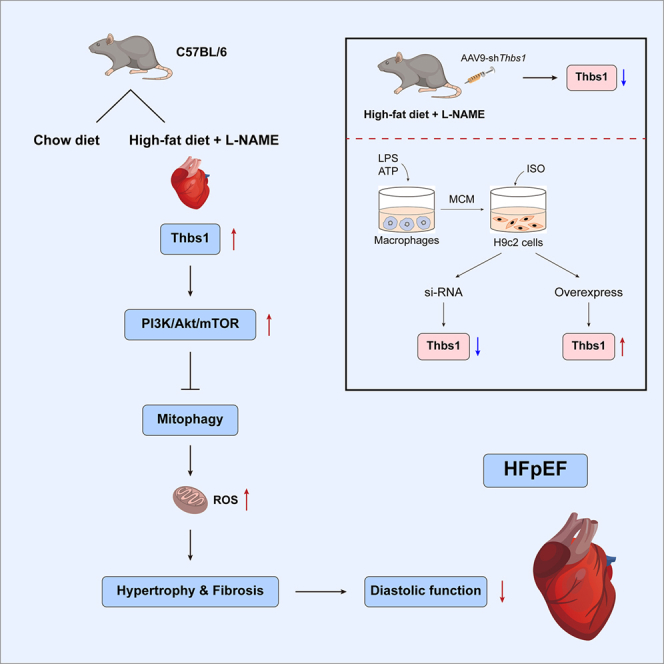

## Introduction

Heart failure with preserved ejection fraction (HFpEF) represents an increasingly prevalent and complex clinical syndrome, accounting for more than half of all heart failure cases, particularly among elderly individuals and those with obesity or metabolic comorbidities.^1^^,^^2^^,^^3^ Despite its high prevalence, efficacious treatment strategies remain lacking, primarily due to the multifactorial and heterogeneous nature of its pathophysiology. Hallmark features of HFpEF include diastolic dysfunction, left ventricular hypertrophy (LVH), interstitial fibrosis, and chronic low-grade inflammation.^4^^,^^5^^,^^6^^,^^7^ These alterations are further compounded by systemic metabolic disturbances, which synergistically contribute to disease onset and progression.

Thrombospondin 1 (Thbs1), a matricellular glycoprotein secreted in response to tissue stress, is known to modulate angiogenesis, extracellular matrix (ECM) remodeling, and immune responses.^8^^,^^9^^,^^10^ While *Thbs1* has been implicated in pathological cardiac hypertrophy and ischemic injury, its specific role in HFpEF has not been clarified.^11^^,^^12^^,^^13^^,^^14^ Given the prominent fibrotic and inflammatory milieu in HFpEF, we hypothesized that *Thbs1* might contribute to disease pathogenesis through modulation of intracellular stress responses.

Mitochondrial dysfunction and defective mitophagy—the selective degradation of damaged mitochondria—are increasingly recognized as critical events in HFpEF.^15^^,^^16^^,^^17^ Cardiomyocytes rely heavily on mitochondrial quality control to maintain energetic and redox balance.^18^^,^^19^ Impaired mitophagy leads to the accumulation of dysfunctional mitochondria, reactive oxygen species (ROS), and inflammatory signals, which collectively exacerbate myocardial dysfunction.^20^^,^^21^ Among the upstream regulators of mitophagy, the phosphatidylinositol 3-kinase (PI3K)/protein kinase B (Akt)/mechanistic target of rapamycin (mTOR) signaling pathway plays a pivotal role in integrating metabolic and stress signals to determine autophagic activity.^22^ Aberrant activation of this pathway not only suppresses autophagic and mitophagic flux but also promotes pathological cardiac remodeling, hypertrophy, and metabolic dysregulation.^23^^,^^24^ However, whether Thbs1 influences HFpEF progression through modulation of the PI3K/Akt/mTOR axis remains unclear.

In this study, we employed a well-established “two-hit” HFpEF mouse model—combining a high-fat diet (HFD) with nitric oxide synthase inhibition (L-NAME)—to investigate the role of *Thbs1* in regulating mitochondrial homeostasis and cardiac remodeling. Using adeno-associated virus (AAV9)-mediated gene knockdown *in vivo* and both loss- and gain-of-function assays in H9c2 cardiomyocytes *in vitro*, we comprehensively examined how Thbs1 influences mitophagy, mitochondrial dynamics, and oxidative stress under HFpEF-like conditions. Integrated transcriptomic and proteomic analyses revealed enrichment of the PI3K/Akt/mTOR signaling pathway in HFpEF myocardium and its positive correlation with *Thbs1* expression. Our findings suggest that *Thbs1* contributes to HFpEF progression by suppressing mitophagy through activation of the PI3K/Akt/mTOR axis, highlighting *Thbs1* as a potential therapeutic target for restoring mitochondrial quality control in HFpEF.

## Results

### “Two-hit” mouse model induces diastolic cardiac dysfunction

**Figure 1:**
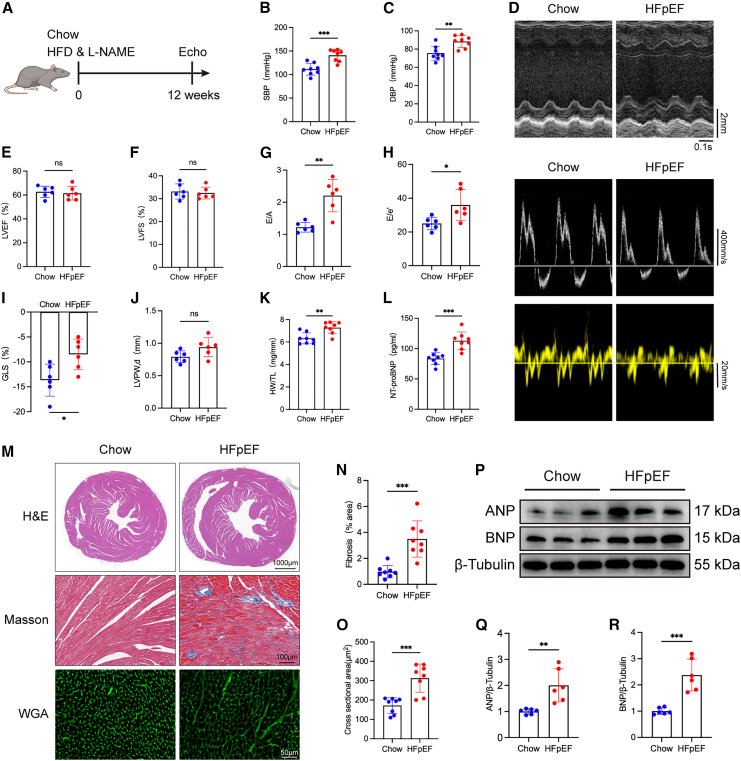
The “two-hit” mouse model recapitulates hallmark features of HFpEF (A) Schematic representation of the experimental protocol. Male C57BL/6 mice (6–8 weeks old) were fed a high-fat diet (HFD) and received L-NAME (0.5 g/L in drinking water) for 12 weeks to induce HFpEF. (B and C) Bar graphs showing systolic blood pressure (SBP) (B) and diastolic blood pressure (DBP) (C), respectively (*n* = 8 biological replicates per group, each measured in triplicate). (D) Representative transthoracic echocardiographic images. (E–J) Quantitative analysis of echocardiographic parameters, including left ventricular ejection fraction (LVEF) (E), fractional shortening (LVFS) (F), E/A ratio (G), E/e′ ratio (H), global longitudinal strain (GLS) (I), and end-diastolic left ventricular posterior wall thickness (LVPWd) (J), (*n* = 6 biological replicates, each averaged from three technical measurements). (K and L) Heart weight-to-tibia length (HW/TL) ratio (K) and plasma NT-proBNP levels (L) (*n* = 8 biological replicates). (M) Representative histological images of hematoxylin-eosin (H&E), Masson’s trichrome, and wheat germ agglutinin (WGA) staining showing cardiomyocyte hypertrophy and interstitial fibrosis. The Scale bars for H&E staining is 1,000 μm, for Masson’s trichrome staining is 100 μm, and for WGA staining is 50 μm. (N and O) Quantification of myocardial fibrotic area (N) and cardiomyocyte cross-sectional area (O) (*n* = 8 biological replicates, each quantified from five random fields per section). (P) Representative western blot images showing hypertrophic markers atrial natriuretic peptide (ANP) and B-type natriuretic peptide (BNP). (Q and R) Densitometric quantification of ANP (Q) and BNP (R) expression normalized to GAPDH (*n* = 6 biological replicates, each from independent tissue lysates). Data are presented as mean ± SD. Statistical significance was determined using an unpaired two-tailed Student’s *t* test for all two-group comparisons. ∗*p* < 0.05, ∗∗*p* < 0.01, ∗∗∗*p* < 0.001, indicating the level of statistical significance for each comparison.

Male C57BL/6 mice (6–8 weeks old) were subjected to HFD combined with L-NAME administration for 12 weeks (Figure 1A). Mice in the HFpEF group developed elevated blood pressure (Figures 1B and 1C). Echocardiographic evaluation revealed hallmark features of HFpEF, including diastolic dysfunction, as evidenced by increased E/A and E/e′ ratios and reduced global longitudinal strain (GLS), while left ventricular ejection fraction (LVEF) and fractional shortening (LVFS) were preserved (Figures 1D–1I). The thickness of the left ventricular posterior wall (LVPW) exhibited an increasing trend (Figure 1J). Additionally, both heart weight and plasma N-terminal pro-B-type natriuretic peptide (NT-proBNP) levels were significantly elevated (Figures 1K and 1L).

Histological examination using Masson’s trichrome and wheat germ agglutinin (WGA) staining demonstrated marked myocardial fibrosis and cardiomyocyte hypertrophy in HFpEF mice (Figures 1M–1O). Western blot analysis further confirmed elevated expression levels of atrial natriuretic peptide (ANP) and BNP (Figures 1P–1R).

Collectively, these findings establish a representative murine HFpEF model characterized by hypertension, diastolic dysfunction, ventricular hypertrophy, and myocardial fibrosis.

### Thbs1 is upregulated in the hearts of HFpEF mice

**Figure 2:**
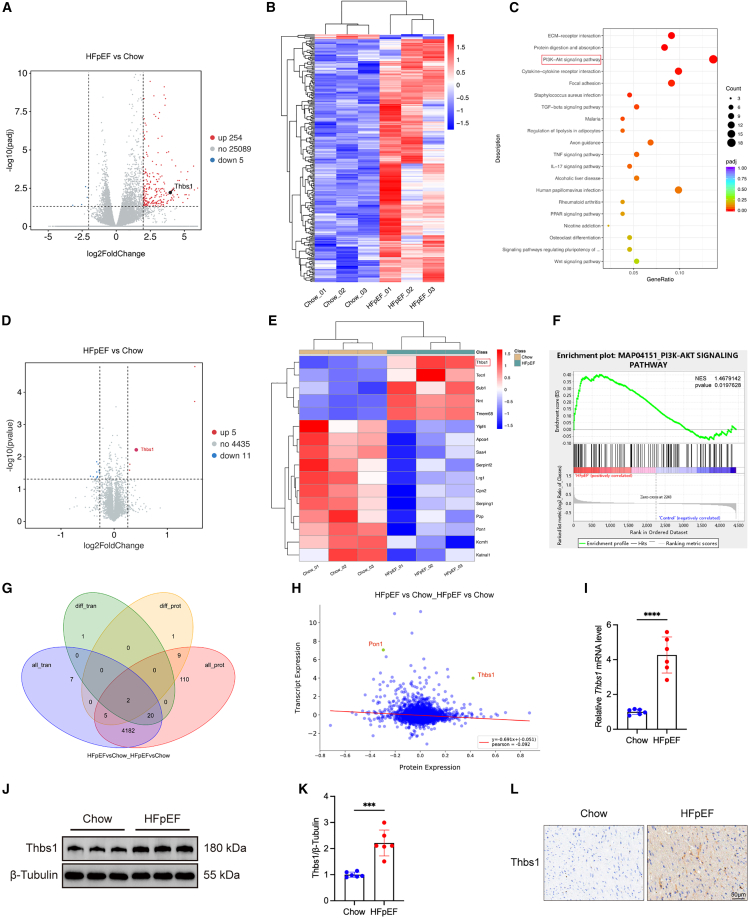
Integrated transcriptomic and proteomic analyses reveal upregulation of *Thbs1* and activation of the PI3K/Akt/mTOR signaling pathway in hearts of HFpEF mice (A) Volcano plot showing differentially expressed genes (DEGs) between HFpEF and Chow mice (|log_2_ fold change| ≥ 2, *p* < 0.05; *n* = 3 biological replicates per group). (B) Heatmap showing expression patterns of DEGs across samples. (C) KEGG pathway enrichment analysis of DEGs. (D) Volcano plot showing differentially expressed proteins (DEPs) from TMT-based proteomics (fold change >1.2 or <0.83, *p* < 0.05; *n* = 3 biological replicates per group). (E) Heatmap of DEPs illustrating group clustering. (F) Gene set enrichment analysis (GSEA) showing activation of the PI3K-Akt signaling pathway at the protein level. (G) Venn diagram showing the overlap between DEGs and DEPs with consistent expression trends. (H) Correlation plot of transcriptomic and proteomic fold changes showing concordance between mRNA and protein expression. (I) Validation of *Thbs1* mRNA upregulation by quantitative reverse-transcription PCR (RT-qPCR) (*n* = 6 biological replicates per group, each measured in triplicate). (J) Representative western blot bands of Thbs1 protein expression. (K) Quantification of Thbs1 protein levels (*n* = 6 biological replicates per group, each measured in triplicate). (L) Immunohistochemical staining of Thbs1 in myocardial sections from Chow and HFpEF mice (Scale bars, 50 μm). Data are presented as mean ± SD. Statistical significance was determined using an unpaired two-tailed Student’s *t* test for two-group comparisons. ∗∗∗*p* < 0.001, ∗∗∗∗*p* < 0.0001, indicating the level of statistical significance for each comparison.

To investigate molecular mechanisms underlying HFpEF, transcriptomic analysis was performed. A total of 259 DEGs were identified in HFpEF hearts relative to chow-fed controls, including 254 upregulated and 5 downregulated genes (|log_2_ fold change| ≥ 2, *p* < 0.05) (Figure 2A). A heatmap illustrating the DEGs is presented in Figure 2B. Kyoto Encyclopedia of Genes and Genomes (KEGG) pathway enrichment analysis revealed significant involvement of ECM-receptor interaction, protein digestion and absorption, PI3K-Akt signaling, cytokine-cytokine receptor interaction, and focal adhesion (Figure 2C).

Proteomic analysis using tandem mass tag (TMT)-based quantitative mass spectrometry identified 16 differentially expressed proteins (DEPs) between HFpEF and control groups, including 11 downregulated and 5 upregulated proteins (fold change >1.2 or <0.83, *p* < 0.05) (Figures 2D and 2E). Gene set enrichment analysis (GSEA) revealed significant activation of the PI3K-Akt pathway at the protein level in HFpEF hearts (Figure 2F).

Integrated transcriptomic and proteomic analyses identified two overlapping genes/proteins with concordant changes (Figure 2G). Among them, Thbs1 was significantly upregulated at both mRNA and protein levels in HFpEF mice, validated by quantitative reverse-transcription PCR (RT-qPCR) and western blotting (Figures 2H–2K). Immunohistochemical staining confirmed enhanced Thbs1 expression localized to both cardiomyocytes and the interstitial space (Figure 2L). Additionally, western blot analysis demonstrated activation of the PI3K/Akt/mTOR pathway in the myocardium of HFpEF mice (Figures S1A–S1D).

### *Thbs1* knockdown attenuates pathological cardiac remodeling in HFpEF mice

To investigate the functional role of *Thbs1 in vivo*, we employed an AAV9, serotype 9 vector (AAV9-cTnT-sh*Thbs1*) designed to achieve cardiomyocyte-specific knockdown of *Thbs1*. The vector expresses a short hairpin RNA embedded within a miR-30 backbone under the control of the cardiac troponin T (cTnT) promoter, ensuring efficient silencing and minimizing systemic or developmental confounders commonly associated with germline knockout models.

Cardiomyocyte specificity of the cTnT promoter was validated using a parallel AAV9-cTnT-EGFP reporter construct, which exhibited restricted EGFP fluorescence in ventricular cardiomyocytes, as shown by immunofluorescence staining of cardiac cryosections (Figure S2A). RT-qPCR and western blot analyses performed 4 weeks post-injection confirmed robust and specific myocardial suppression of Thbs1 expression (Figures S2B–S2D).

**Figure 3:**
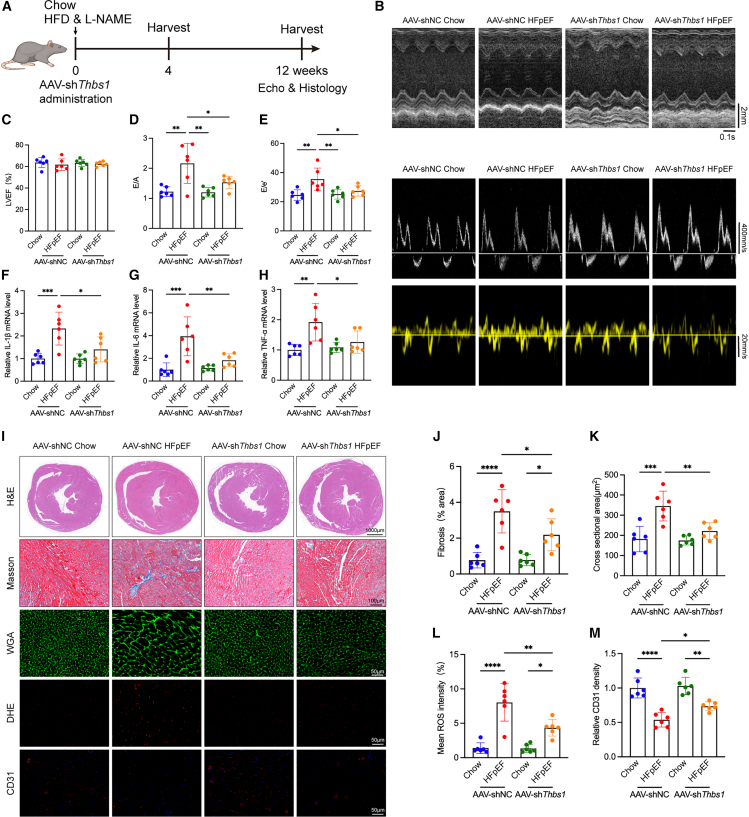
Cardiac-specific knockdown of *Thbs1* alleviates diastolic dysfunction, myocardial fibrosis, and oxidative stress in HFpEF mice (A) Experimental timeline showing that AAV9-mediated *Thbs1* knockdown (AAV9-cTnT-sh*Thbs1* or AAV9-NC) and HFpEF induction by HFD combined with L-NAME were initiated concurrently and maintained for 12 weeks. (B) Representative echocardiographic images illustrating cardiac morphology and diastolic function. (C–E) Quantitative analysis of echocardiographic parameters, including (C) left ventricular ejection fraction (LVEF), (D) E/A ratio, and (E) E/e′ ratio (*n* = 6 biological replicates per group, each measured in triplicate). (F–H) RT-qPCR analysis of pro-inflammatory cytokine mRNA levels, including IL-1β (F), IL-6 (G), and TNF-α (H) (*n* = 6 biological replicates per group, each measured in duplicate). (I) Representative histological and immunofluorescent images showing H&E staining for overall morphology, Masson’s trichrome staining for fibrosis, WGA staining for cardiomyocyte size, DHE staining for reactive oxygen species (ROS), and CD31 immunofluorescence for capillary density. The Scale bars for H&E staining is 1,000 μm, for Masson’s trichrome, staining is 100 μm, for WGA, staining is 50 μm, for DHE, staining is 50 μm, and for CD31, staining is 50 μm. (J–M) Quantification of histological parameters: (J) myocardial fibrosis area from Masson’s staining, (K) cardiomyocyte cross-sectional area from WGA staining, (L) mean ROS fluorescence intensity from DHE staining, and (M) capillary density from CD31 staining (*n* = 6 biological replicates per group). Data are presented as mean ± SD. Statistical significance was determined using one-way ANOVA followed by Tukey’s post hoc test. ∗*p* < 0.05, ∗∗*p* < 0.01, ∗∗∗*p* < 0.001, ∗∗∗∗*p* < 0.0001, indicating the level of statistical significance for each comparison.

Following AAV administration, mice were simultaneously subjected to HFD and L-NAME treatment to induce the HFpEF phenotype (Figure 3A). This parallel design ensured that *Thbs1* knockdown occurred throughout the development of HFpEF. Systolic blood pressure did not differ significantly between AAV9-cTnT-sh*Thbs1* and AAV9-NC mice under either Chow or HFpEF conditions, suggesting that the cardioprotective effects observed in the HFpEF + sh*Thbs1* group were not secondary to systemic hemodynamic alterations (Figures S3A and S3B). Echocardiography revealed that *Thbs1* knockdown preserved LVEF but significantly improved diastolic function, as reflected by reduced E/A and E/e′ ratios and improved GLS (Figures 3B–3E and S3C). Heart weight was also reduced, indicating attenuation of hypertrophy (Figure S3D).

HFpEF is associated with inflammation and oxidative stress. *Thbs1* knockdown reduced myocardial expression of pro-inflammatory cytokines interleukin (IL)-1β, IL-6, and tumor necrosis factor-alpha (TNF-α) (Figures 3F–3H). Masson’s and WGA staining showed diminished fibrosis and hypertrophy. Dihydroethidium (DHE) staining indicated reduced ROS levels, and CD31 immunostaining revealed improved capillary density in AAV-sh*Thbs1*-treated hearts (Figures 3I–3M).

### *Thbs1* knockdown partially restores mitophagy in HFpEF mice

**Figure 4:**
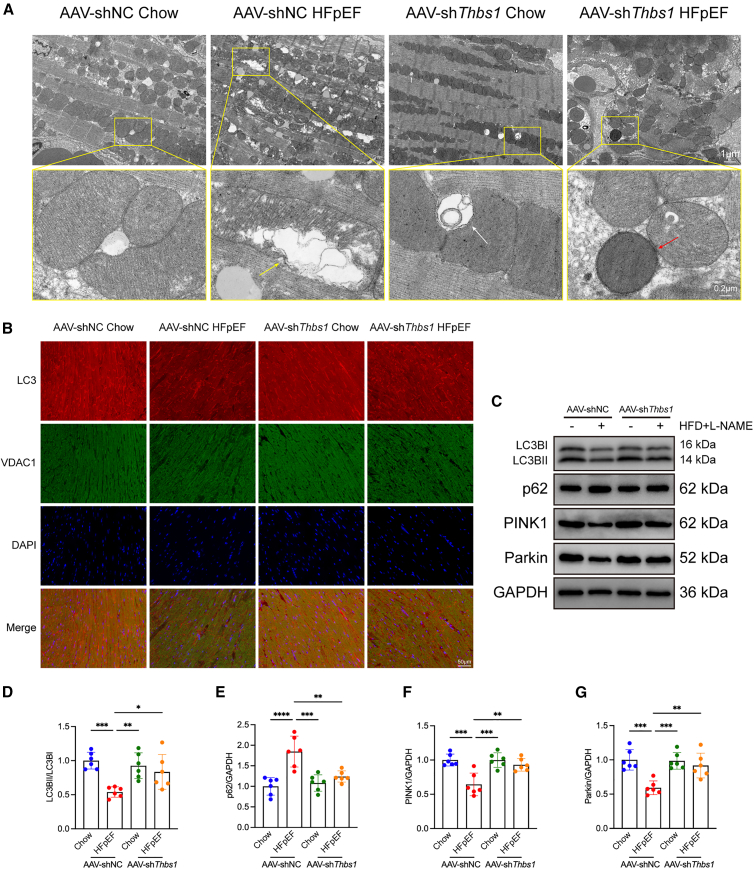
*Thbs1* knockdown restores mitochondrial morphology and mitophagy in hearts of HFpEF mice (A) Transmission electron microscopy (TEM) images of left ventricular myocardium. Hearts from AAV9-shNC HFpEF mice displayed disrupted mitochondrial morphology, including swollen mitochondria and fragmented cristae (yellow arrows). *Thbs1* knockdown partially restored mitochondrial structure and enhanced autophagic activity, as evidenced by increased mitophagosome formation (red arrows). Secondary lysosomes are indicated by white arrows. The Scale bars for the first row is 1 μm, and for the second row (magnified view), the Scale bars represent 0.2 μm. (B) Immunofluorescence staining of LC3 (red) and VDAC (green) in myocardial sections, showing reduced LC3-mitochondria colocalization in HFpEF hearts and restoration upon *Thbs1* knockdown. Scale bars, 50 μm. (C) Representative western blot bands of mitophagy-related proteins LC3-II/I, p62, PINK1, and Parkin in heart tissues. (D–G) Quantification of protein expression: LC3-II/I, (D) p62 (E) PINK1, (F) and Parkin (G) (*n* = 6 biological replicates per group, each measured in duplicate). Data are presented as mean ± SD. Statistical significance was determined by one-way ANOVA followed by Tukey’s post hoc test. ∗*p* < 0.05, ∗∗*p* < 0.01, ∗∗∗*p* < 0.001, ∗∗∗∗*p* < 0.0001, indicating the level of statistical significance for each comparison.

Transmission electron microscopy (TEM) revealed that HFpEF induced mitochondrial fragmentation and reduced mitophagic vesicles, while *Thbs1* knockdown partially restored mitochondrial morphology and autophagosome formation (Figure 4A).

Immunofluorescence demonstrated reduced LC3 localization to voltage-dependent anion channel (VDAC)-labeled mitochondria in HFpEF mice, which was rescued by *Thbs1* knockdown (Figure 4B). Western blot analysis showed that *Thbs1* silencing restored LC3-II/I, PINK1, and Parkin expression and reduced p62 accumulation (Figures 4C–4G).

### *Thbs1* bidirectionally regulates cardiomyocyte injury and mitophagy *in vitro*

**Figure 5:**
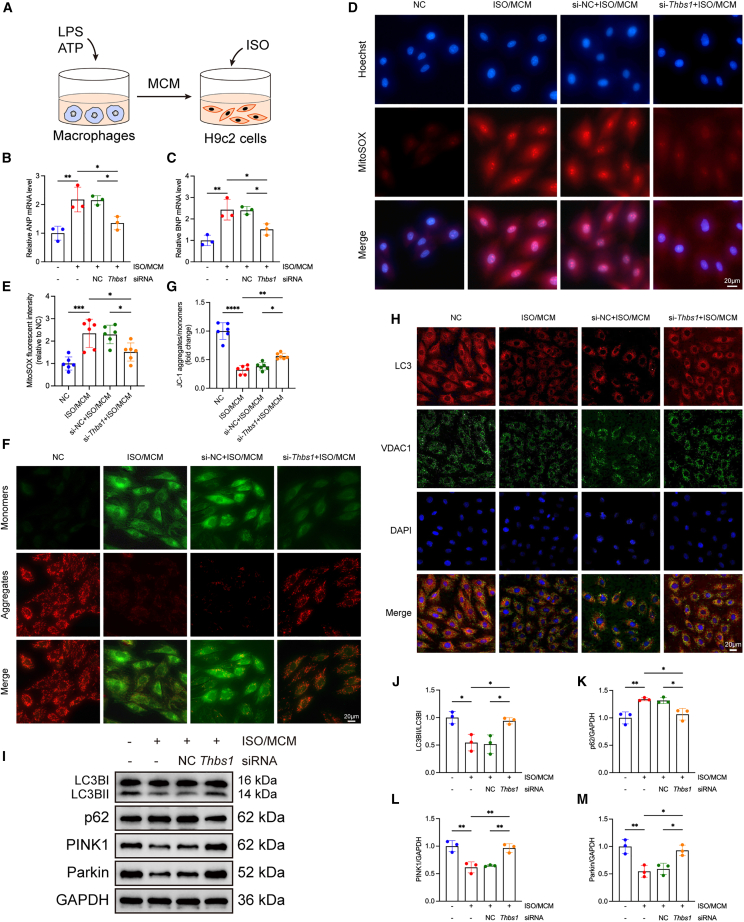
*Thbs1* knockdown alleviates cardiomyocyte hypertrophy, oxidative stress, mitochondrial dysfunction, and mitophagy impairment *in vitro* (A) Schematic diagram of the *in vitro* pathological stimulation protocol. H9c2 cells were treated with isoproterenol (ISO) and macrophage-conditioned medium (MCM) derived from LPS+ATP-activated macrophages, with or without *Thbs1* knockdown. (B and C) mRNA levels of hypertrophic markers ANP (B) and BNP (C) under pathological stimulation (*n* = 3 biological replicates per group, each measured in triplicate). (D) Representative fluorescence images of mitochondrial reactive oxygen species (ROS) detected using MitoSOX Red. Scale bars, 20 μm. (E) Quantification of MitoSOX Red fluorescence intensity (*n* = 6 biological replicates per group, each measured in triplicate). (F) JC-1 staining to assess mitochondrial membrane potential (MMP), representative images shown. Scale bars, 20 μm. (G) Quantification of JC-1 aggregates/monomers ratio (*n* = 6 biological replicates per group, each measured in triplicate). (H) Immunofluorescence images showing LC3 (red) and VDAC (green) colocalization; nuclei were stained with DAPI (blue). Scale bars, 20 μm. (I) Representative western blot images of mitophagy-related proteins LC3, p62, PINK1, and Parkin. (J–M) Quantification of protein expression: LC3-II/I (J), p62 (K), PINK1 (L), and Parkin (M) (*n* = 3 biological replicates per group, each measured in duplicate). Data are presented as mean ± SD. Statistical significance was determined by one-way ANOVA followed by Tukey’s post hoc test. ∗*p* < 0.05, ∗∗*p* < 0.01, ∗∗∗*p* < 0.001, ∗∗∗∗*p* < 0.0001, indicating the level of statistical significance for each comparison.

To investigate the role of *Thbs1* in cardiomyocyte injury under pathological stress, we established an *in vitro* model by exposing H9c2 cells to isoproterenol (ISO) and macrophage-conditioned medium (MCM) derived from NLRP3 inflammasome-activated macrophages, thereby recapitulating the inflammatory and neurohumoral milieu characteristic of HFpEF-related cardiac stress (Figure 5A).

Loss-of-function experiments were initially conducted using siRNA-mediated knockdown of *Thbs1*. Three siRNA sequences targeting *Thbs1* were screened, and their silencing efficiency was evaluated by RT-qPCR. Among these, si-*Thbs1*-1 showed the most efficient knockdown at the mRNA level (Figure S4A), and thus, si-*Thbs1*-1 was selected for all subsequent experiments. Protein-level knockdown of Thbs1 was confirmed via western blot analysis (Figures S4B and S4C). Notably, *Thbs1* knockdown significantly attenuated the increase in hypertrophic markers ANP and BNP induced by ISO and MCM (Figures 5B and 5C) and alleviated cellular hypertrophy, as demonstrated by Phalloidin-TRITC staining (Figures S5A and S5B). Additionally, *Thbs1* silencing significantly reduced intracellular ROS production (Figures 5D and 5E) and restored MMP, as assessed by JC-1 staining (Figures 5F and 5G). Immunofluorescence analysis further revealed enhanced colocalization of LC3 with the mitochondrial marker VDAC in the knockdown group, suggesting partial restoration of mitophagy (Figure 5H). Western blot analysis showed that *Thbs1* knockdown restored the expression of key mitophagy regulators, including LC3-II/I, PINK1, and Parkin, while reducing p62 accumulation (Figures 5I–5M). These findings suggest that Thbs1 suppression confers protection against stress-induced cardiomyocyte injury by mitigating hypertrophy, oxidative stress, mitochondrial dysfunction, and impaired mitophagy.

**Figure 6:**
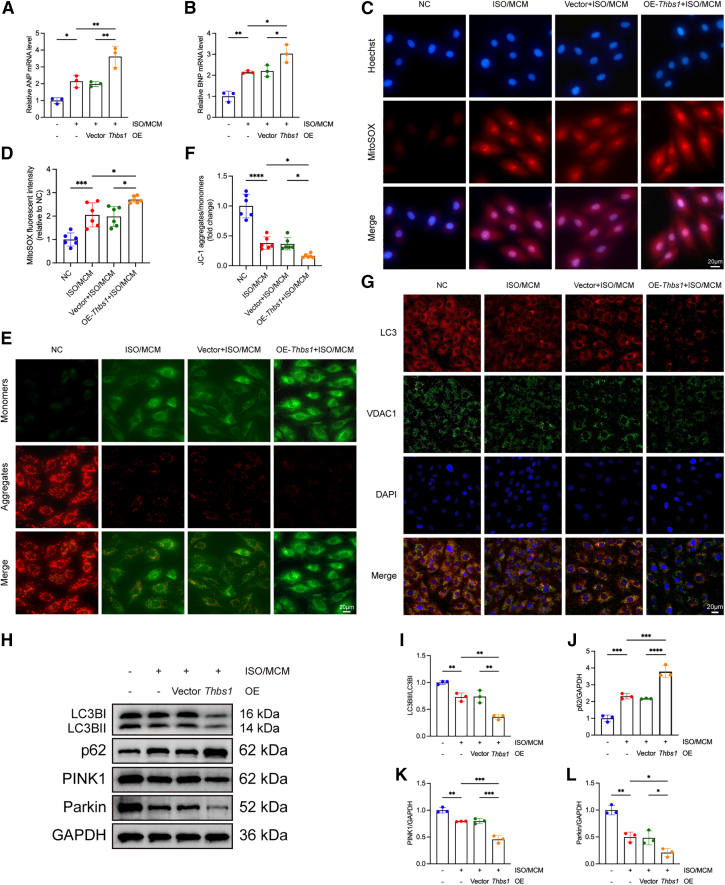
*Thbs1* overexpression exacerbates cardiomyocyte hypertrophy, oxidative stress, mitochondrial dysfunction, and mitophagy impairment under pathological stimulation (A and B) mRNA expression of hypertrophic markers ANP (A) and BNP (B) under pathological stimulation with or without *Thbs1* overexpression (*n* = 3 biological replicates per group, each measured in triplicate). (C) Representative fluorescence images of mitochondrial ROS detected using MitoSOX Red. Scale bars, 20 μm. (D) Quantification of MitoSOX Red fluorescence intensity (*n* = 6 biological replicates per group, each measured in triplicate). (E) Representative JC-1 staining images reflecting MMP. Scale bars, 20 μm. (F) Quantification of JC-1 aggregates/monomers ratio (*n* = 6 biological replicates per group, each measured in triplicate). (G) Immunofluorescence images showing LC3 (red) and VDAC (green) colocalization; nuclei were stained with DAPI (blue). Scale bars, 20 μm. (H) Representative western blot images of mitophagy-related proteins LC3, p62, PINK1, and Parkin. (I–L) Quantitative of protein expression: LC3-II/I (I), p62 (J), PINK1 (K), and Parkin (L) (*n* = 3 biological replicates per group, each measured in duplicate). Data are presented as mean ± SD. Statistical significance was determined by one-way ANOVA followed by Tukey’s post hoc test. ∗*p* < 0.05, ∗∗*p* < 0.01, ∗∗∗*p* < 0.001, ∗∗∗∗*p* < 0.0001, indicating the level of statistical significance for each comparison.

To further validate these findings, gain-of-function experiments were performed by overexpressing *Thbs1* in H9c2 cells. Successful overexpression was confirmed at both the transcript and protein levels (Figures S6A–S6C). Compared to vector controls, *Thbs1*-overexpressing cells showed significantly increased expression of ANP and BNP (Figures 6A and 6B), along with more pronounced cellular hypertrophy (Figures S7A and S7B) upon stimulation with ISO and MCM. In parallel, *Thbs1* overexpression further exacerbated ROS production (Figures 6C and 6D), MMP loss (Figures 6E and 6F), and mitophagy impairment, as indicated by reduced LC3-VDAC colocalization (Figure 6G). Western blot analysis revealed decreased levels of LC3-II/I, PINK1, and Parkin, along with increased p62 accumulation, suggesting suppression of mitophagic flux (Figures 6H–6L).

Taken together, these bidirectional functional studies highlight the central role of *Thbs1* in regulating cardiomyocyte responses to pathological stress. While *Thbs1* knockdown alleviates hypertrophy, oxidative damage, and mitophagy dysfunction, its overexpression exacerbates these detrimental effects. These findings underscore *Thbs1* as a critical mediator and potential therapeutic target for stress-induced cardiac injury.

### *Thbs1* regulates mitophagy in H9c2 cardiomyocytes via ITGB1-mediated PI3K/Akt/mTOR signaling

**Figure 7:**
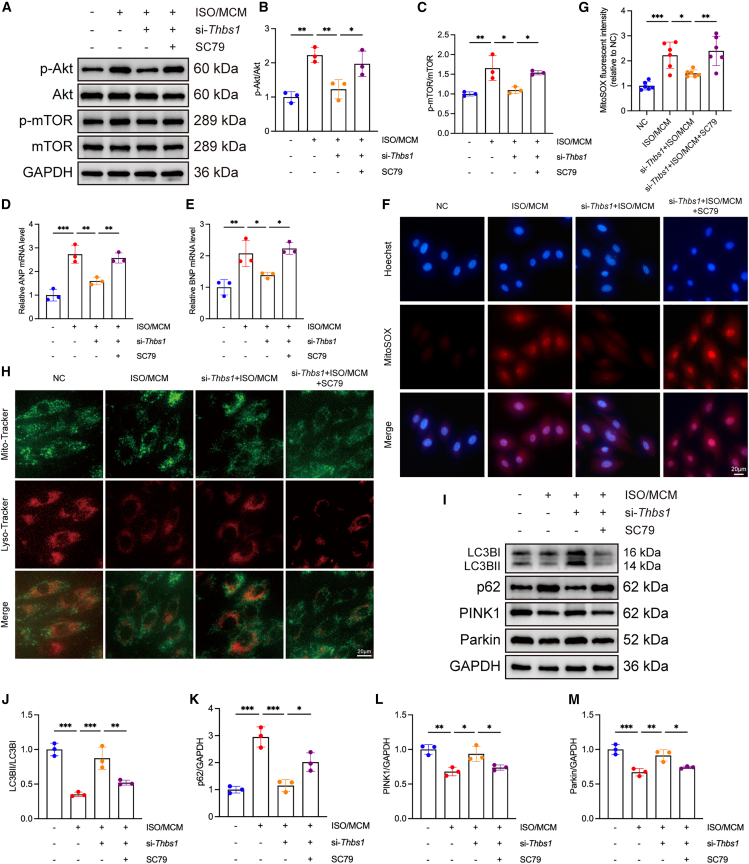
PI3K/Akt/mTOR pathway activation reverses the protective effects of *Thbs1* knockdown in H9c2 cells (A) Representative western blot images showing phosphorylation and total levels of Akt and mTOR in H9c2 cells transfected with si-*Thbs1* and treated with ISO plus MCM or the Akt activator SC79. (B and C) Quantification of protein phosphorylation: *p*-Akt/Akt (B) and *p*-mTOR/mTOR (C) (*n* = 3 biological replicates per group, each measured in duplicate). (D and E) Relative mRNA expression of ANP (D) and BNP (E) assessed by RT-qPCR (*n* = 3 biological replicates per group, each measured in triplicate). (F and G) Intracellular ROS production measured using MitoSOX Red mitochondrial superoxide indicator; representative fluorescence images are shown (F) and quantified as mean fluorescence intensity (G) (*n* = 6 biological replicates per group, each measured in triplicate). Scale bars, 20 μm. (H) Representative confocal images of mitochondria-lysosome colocalization using Mito-Tracker (green) and Lyso-Tracker (red). Scale bars, 20 μm. (I) Representative western blot images of mitophagy-related proteins LC3, p62, PINK1, and Parkin. (J–M) Quantification of mitophagy-related protein expression: LC3-II/I (J), p62 (K), PINK1 (L), and Parkin (M) (*n* = 3 biological replicates per group, each measured in duplicate). Data are presented as mean ± SD. Statistical significance was determined by one-way ANOVA followed by Tukey’s post hoc test. ∗*p* < 0.05, ∗∗*p* < 0.01, ∗∗∗*p* < 0.001, indicating the level of statistical significance for each comparison.

Transcriptomic and proteomic analyses indicated that the PI3K-Akt signaling pathway may play a crucial role in the pathogenesis of HFpEF. To validate this further, western blot analysis demonstrated that *Thbs1* knockdown inhibited the activation of the PI3K/Akt/mTOR pathway, both *in vivo* and *in vitro* models (Figures S8A–S8D; Figures 7A–7C).

To determine whether the protective effects of *Thbs1* knockdown are mediated through inhibition of the Akt pathway, we applied the Akt-specific activator SC79 to H9c2 cells transfected with si-*Thbs1* under ISO and MCM stimulation. Western blot analysis confirmed that SC79 treatment restored the phosphorylation of Akt and its downstream mTOR signaling components (Figures 7A–7C). Functionally, SC79 treatment reversed the reductions in ANP, BNP, and ROS levels induced by *Thbs1* knockdown (Figures 7D–7G). Furthermore, MitoTracker and LysoTracker colocalization assays revealed that *Thbs1* knockdown markedly enhanced mitochondria-lysosome colocalization, indicating increased mitophagic activity, whereas SC79 treatment attenuated this effect (Figure 7H). Consistently, western blot analysis showed that SC79 treatment decreased LC3-II/I, PINK1, and Parkin expression while increasing p62 accumulation, suggesting that Akt/mTOR pathway activation counteracted the mitophagy enhancement caused by *Thbs1* knockdown (Figures 7I–7M).

**Figure 8:**
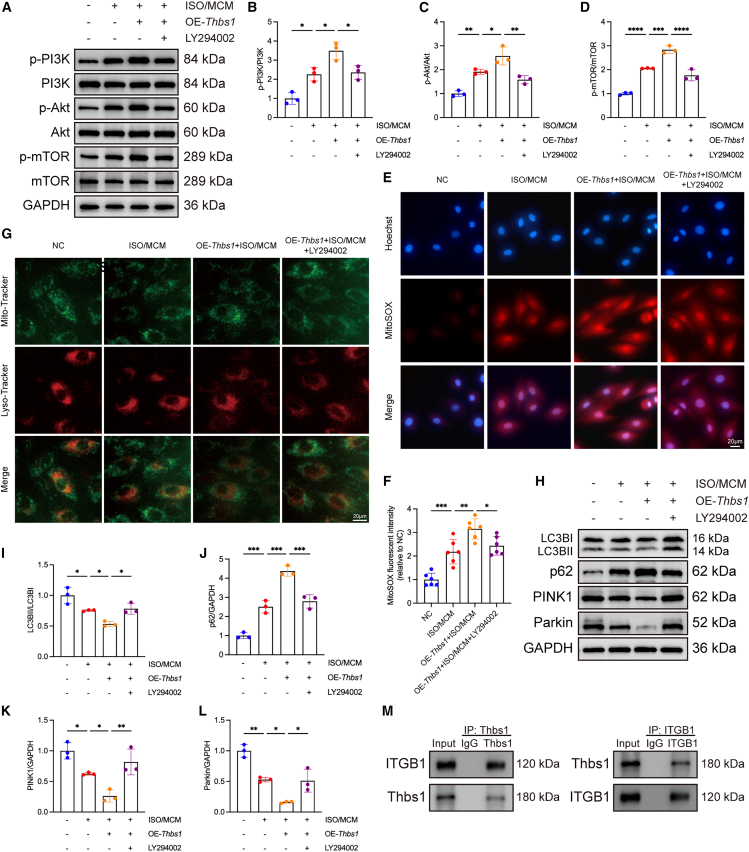
Inhibition of PI3K/Akt/mTOR reverses effects of *Thbs1* overexpression and Thbs1 binds ITGB1 in H9c2 cells (A) Representative western blot images showing phosphorylation and total levels of PI3K, Akt, and mTOR in H9c2 cells transfected with *Thbs1* overexpression plasmid and treated with ISO plus MCM or the PI3K/Akt/mTOR inhibitor LY294002. (B–D) Quantification of protein phosphorylation: p-PI3K/PI3K (B), *p*-Akt/Akt (C), and *p*-mTOR/mTOR (D) (*n* = 3 biological replicates per group, each measured in duplicate). (E and F) Intracellular ROS production measured using MitoSOX Red; representative fluorescence images are shown (E) and quantified as mean fluorescence intensity (F) (*n* = 6 biological replicates per group, each measured in triplicate). Scale bars, 20 μm. (G) Representative confocal images showing mitochondria-lysosome colocalization using Mito-Tracker (green) and Lyso-Tracker (red). Scale bars, 20 μm. (H) Representative western blot images of mitophagy-related proteins LC3, p62, PINK1, and Parkin. (I–L) Quantification of mitophagy-related protein expression: LC3-II/I (I), p62 (J), PINK1 (K), and Parkin (L) (*n* = 3 biological replicates per group, each measured in duplicate). (M) Co-immunoprecipitation (Co/IP) assays showing the interaction between Thbs1 and ITGB1 in H9c2 cells. Data are presented as mean ± SD. Statistical significance was determined by one-way ANOVA followed by Tukey’s post hoc test. ∗*p* < 0.05, ∗∗*p* < 0.01, ∗∗∗*p* < 0.001, ∗∗∗∗*p* < 0.0001, indicating the level of statistical significance for each comparison.

In contrast, western blot analysis showed that *Thbs1* overexpression strongly activated the PI3K/Akt/mTOR signaling pathway in H9c2 cells exposed to ISO and MCM, compared with control cells (Figures 8A–8D). To investigate whether inhibiting this pathway could mitigate the detrimental effects of *Thbs1* overexpression, we utilized the PI3K inhibitor LY294002. Western blot analysis confirmed effective suppression of the PI3K/Akt/mTOR axis upon LY294002 treatment (Figures 8A–8D). Functionally, LY294002 treatment alleviated the *Thbs1*-induced increases in ANP, BNP, and ROS levels (Figures S9A and S9B; Figures 8E and 8F) and partially restored mitophagy, as evidenced by increased mitochondria-lysosome colocalization (Figure 8G) and elevated LC3-II/I, PINK1, and Parkin levels with concomitant reduction in p62 accumulation (Figures 8H–8L).

In summary, these results indicate that *Thbs1* modulates mitophagy in H9c2 cardiomyocytes via the PI3K/Akt/mTOR signaling pathway: knockdown enhances mitophagy by suppressing this signaling cascade, whereas overexpression inhibits mitophagy through pathway activation.

Finally, to address the upstream receptor mediating Thbs1 signaling, we performed co-immunoprecipitation (Co-IP) in H9c2 cells and found that Thbs1 physically interacts with integrin β1 (ITGB1) (Figure 8M), suggesting that ITGB1 serves as a functional receptor linking Thbs1 to activation of the PI3K/Akt/mTOR axis.

## Discussion

In this study, we provide compelling evidence that *Thbs1* plays a central role in the pathogenesis of HFpEF by modulating mitophagy and mitochondrial homeostasis via the PI3K/Akt/mTOR signaling pathway. Using a well-characterized “two-hit” HFpEF mouse model and *in vitro* assays, we show that Thbs1 protein is significantly upregulated in the HFpEF myocardium, and that *Thbs1* knockdown ameliorates multiple pathological features, including cardiac hypertrophy, fibrosis, oxidative stress, and mitochondrial dysfunction. These findings position Thbs1 not only as a biomarker of cardiac stress but also as an active mediator of maladaptive remodeling in HFpEF.

Thbs1 is a multifunctional matricellular protein involved in ECM remodeling, inflammation, and angiogenesis, with context-dependent effects in preclinical cardiac models.^25^^,^^26^^,^^27^^,^^28^^,^^29^ Knockout of *Thbs1* can lead to early hypertrophy and late ventricular dilation, whereas overexpression may cause lethal cardiac atrophy.^13^^,^^30^^,^^31^ These phenotypes likely reflect Thbs1’s interactions with multiple receptors, including α9β1 integrin, CD36, and CD47.^32^^,^^33^^,^^34^ Beyond classical remodeling, Thbs1 has been implicated in cardiorenal syndrome and chronic kidney disease-related LVH, mediating myocardial remodeling and inflammation through transforming growth factor-β1, extracellular signal-regulated kinase (ERK), and aryl hydrocarbon receptor pathways, and promoting oxidative stress and cellular senescence.^35^ In this study, we identify an important role of *Thbs1* in HFpEF as a context-dependent mediator of maladaptive remodeling through suppression of mitophagy, highlighting mitochondrial quality control as a potential therapeutic target.

Mitophagy, a selective form of autophagy that removes damaged mitochondria, is critical for maintaining mitochondrial integrity under stress conditions such as metabolic overload or oxidative injury.^20^^,^^36^^,^^37^^,^^38^^,^^39^^,^^40^^,^^41^ In our model, *Thbs1* knockdown enhanced mitochondrial LC3 recruitment, restored autophagic flux, and reduced mitochondrial ROS production, indicating protective effects via mitophagy restoration. Mechanistically, *Thbs1* knockdown inhibited the PI3K/Akt/mTOR pathway, a known negative regulator of autophagy,^42^^,^^43^^,^^44^ whereas *Thbs1* overexpression activated this axis. Transcriptomic and proteomic profiling confirmed enrichment of the PI3K/Akt/mTOR pathway in HFpEF hearts, and co-immunoprecipitation demonstrated that *Thbs1* physically interacts with ITGB1, suggesting that ITGB1 functions as a receptor linking Thbs1 to PI3K/Akt/mTOR activation. Collectively, these findings indicate that *Thbs1* integrates multiple extracellular stress cues—including inflammatory, mechanical, and oxidative signals—through receptor-mediated PI3K/Akt/mTOR activation, impairing mitochondrial quality control and driving maladaptive remodeling.

Emerging evidence suggests that Thbs1 mediates crosstalk among cardiomyocytes, immune cells, and fibroblasts. As a secreted matricellular protein, Thbs1 can act in a paracrine manner to amplify inflammatory and fibrotic responses: in cardiomyocytes, it suppresses mitophagy and increases oxidative stress; in fibroblasts, it promotes ECM deposition; and in immune cells, it contributes to low-grade chronic inflammation characteristic of HFpEF.^45^^,^^46^^,^^47^ These combined effects position Thbs1 as a central mediator co-ordinating systemic maladaptive remodeling in HFpEF.

The therapeutic potential of *Thbs1* is substantial. Its secreted nature makes Thbs1 an attractive candidate for clinical translation, as serum levels could serve as a biomarker of disease progression, cardiac stress, and therapeutic response. Ongoing clinical work in our laboratory is assessing serum Thbs1 in HFpEF patients compared with age-matched controls to explore associations with echocardiographic parameters, cardiac biomarkers, and disease severity. While these data are not yet included, they highlight the translational potential of *Thbs1*.

Finally, *Thbs1* inhibition or autophagy restoration via mTOR modulation represents a promising therapeutic strategy. In our study, *Thbs1* knockdown reduced fibrosis, hypertrophy, and oxidative stress, suggesting that targeting the Thbs1-PI3K/Akt/mTOR axis could restore mitochondrial function and improve cardiac performance in HFpEF.

In conclusion, this study establishes *Thbs1* as a pivotal regulator of mitochondrial quality control in HFpEF. Mechanistically, *Thbs1* impairs mitophagy through activation of PI3K/Akt/mTOR signaling, contributing to oxidative stress and maladaptive remodeling. Targeting *Thbs1* offers a potential avenue for therapeutic intervention in HFpEF, a condition with limited effective treatments.

### Limitations of the study

While this study provides significant insights into the role of *Thbs1* in HFpEF pathogenesis, several limitations should be noted. First, although the “two-hit” HFpEF mouse model, combining HFD with L-NAME administration, effectively replicates key features of human HFpEF, it does not fully capture the clinical heterogeneity observed in patients, particularly those with comorbidities, such as diabetes, aging, or atrial fibrillation. In our experiments, AAV9-mediated *Thbs1* knockdown was administered at the onset of HFpEF induction. Based on established kinetics, AAV9-mediated knockdown reaches maximal efficiency, approximately 3–4 weeks post-injection and maintains sustained suppression throughout the 12-week modeling period, as confirmed by mRNA and protein analysis. We recognize that this design has a preventive component, and future studies initiating *Thbs1* knockdown after HFpEF establishment will be necessary to assess therapeutic efficacy and better mimic the clinical scenario.

Second, *in vitro* experiments employed H9c2 cardiomyoblasts exposed to MCM from NLRP3 inflammasome-activated macrophages, together with ISO stimulation, to simulate the combined inflammatory and neurohumoral stress characteristic of HFpEF. While this model has been validated in previous HFpEF studies, it represents a simplified system that cannot fully recapitulate the chronic, low-grade, multicellular inflammatory milieu in patients. Moreover, H9c2 cells exhibit immature electrophysiological and metabolic properties compared with adult cardiomyocytes, particularly regarding mitochondrial function and oxidative metabolism. Future studies using more physiologically relevant models—such as primary adult cardiomyocytes, co-culture systems integrating macrophages and cardiomyocytes, or human induced pluripotent stem cell (iPSC)-derived cardiomyocytes—would further strengthen the translational relevance of *Thbs1*-mediated mitochondrial and autophagic regulation.

Finally, although transcriptomic and proteomic analyses of HFpEF myocardium revealed enrichment of the PI3K/Akt/mTOR signaling pathway, multi-omics integration at the single-cell or spatial level was not performed. Single-cell approaches could provide more precise insights into cell-type-specific mechanisms of *Thbs1*-mediated mitophagy dysregulation, allowing the identification of distinct cardiomyocyte subpopulations and non-cardiomyocyte cell types that contribute to HFpEF pathology, and thereby improving the precision of potential therapeutic targeting.

## Resource availability

### Lead contact

Requests for further information and resources should be directed to and will be fulfilled by the lead contact, Huanzhen Chen (chenhz@sxmu.edu.cn).

### Materials availability

This study did not generate new unique reagents.

### Data and code availability

• RNA sequencing (RNA-seq) data generated in this study have been deposited in the Gene Expression Omnibus (GEO; https://www.ncbi.nlm.nih.gov/geo/) under the public accession number GSE305470 and are fully accessible without restriction.
• Proteomic data generated in this study have been deposited in the Integrated Proteome Resources (iProX; https://www.iprox.cn) under the public accession number IPX0013041000 and are fully accessible without restriction.
• This paper does not report original code.
• Any additional information required to reanalyze the data reported in this paper is available from the lead contact upon request.

## Acknowledgments

This work was supported by the Fundamental Research Program of Shanxi Province (202403021221298 and 202203021211068) and the Traditional Chinese Medicine Research Project of Shanxi Province (2024ZYY2C041).

## Author contributions

Conceptualization, H.C.; methodology, X.B., S.S., Z.Z., S.B., and S.F.; writing – original draft, H.C. and X.B.; writing – review and editing, H.C., X.B., S.S., Z.Z., and S.F.; funding acquisition, H.C., X.B., and L.W.; resources, S.F., C.L., and L.W.; data curation, S.B., C.L., and L.W.; supervision, H.C.

## Declaration of interests

The authors declare no competing interests.

## Declaration of generative AI and AI-assisted technologies in the writing process

The authors did not use generative AI or AI-assisted technologies in the development of this manuscript.

## STAR★Methods

### Key resources table

**Table:** 

REAGENT or RESOURCE	SOURCE	IDENTIFIER
**Antibodies**		

anti-ANP	Boster Biological Technology	Cat# A01318-1; RRID: N/A
anti-BNP	Abmart	Cat# PS00154; RRID: N/A
anti-Thbs1	Abcam	Cat# ab267388; RRID: AB_3271579
anti-LC3B	Abways	Cat# CY5992; RRID: N/A
anti-p62	Abways Technology	Cat# CY5546; RRID: AB_3714778
anti-PINK1	Cell Signaling Technology	Cat# 6946; RRID: AB_11179069
anti-Parkin	Abways Technology	Cat# CY6641; RRID: N/A
anti-p-PI3K	Abways Technology	Cat# CY6427; RRID: AB_3099429
anti-PI3K	Abways Technology	Cat# CY6915; RRID: AB_3665436
anti-*p*-Akt	Abways Technology	Cat# CY6569; RRID: AB_3101809
anti-Akt	Abways Technology	Cat# CY5561; RRID: AB_3099435
anti-*p*-mTOR	Abways Technology	Cat# CY6571; RRID: AB_3099434
anti-mTOR	Abways Technology	Cat# CY5306; RRID: AB_3099432
anti-β-Tubulin	Abways Technology	Cat# AB0039; RRID: AB_2904143
anti-GAPDH	Abways Technology	Cat# AB0037; RRID: AB_2891315
Goat Anti-Rabbit IgG H&L (HRP)	Abcam	Cat# ab6721; RRID: AB_955447
Anti-LC3	Santa Cruz Biotechnology	Cat# sc-271625; RRID: AB_10714949
Anti-VDAC1	ServiceBio	Cat#GB111939; RRID: N/A
Goat Anti-Mouse IgG (H + L) Antibody, Secondary Antibody, Cy3-conjugated	ServiceBio	Cat# GB21301; RRID: AB_2923552
Goat Anti-Rabbit IgG (H + L) Antibody, Secondary Antibody, Alexa Fluor® 488-conjugated	ServiceBio	Cat# GB25303; RRID: AB_2910224
Anti-integrin β1/ITGB1 (A-4)	Santa Cruz Biotechnology	Cat# sc-374429; RRID: AB_11012020
Rabbit IgG, monoclonal [EPR25A] - Isotype Control	Abcam	Cat# ab172730; RRID: AB_2687931
Mouse IgG1, kappa monoclonal [MOPC-21] - isotype control	Abcam	Cat# ab18443; RRID: AB_2736846
Goat Anti-Mouse IgG H&L (HRP)	Abcam	Cat# ab205719; RRID: AB_2755049

**Bacterial and virus strains**		

AAV9-cTnT-shThbs1	Hanbio Biotechnology	Custom construct
AAV9-cTnT-shScramble (control)	Hanbio Biotechnology	Custom construct
AAV9-cTnT-EGFP (reporter)	Hanbio Biotechnology	Custom construct

**Chemicals, peptides, and recombinant proteins**		

L-NAME	Sigma-Aldrich	Cat# N5751
High-fat diet (60% kcal fat)	Research Diets	Cat# D12492
Lipopolysaccharide (LPS)	Sigma-Aldrich	Cat# L4391
Adenosine triphosphate (ATP)	Sigma-Aldrich	Cat# A6559
Isoproterenol (ISO)	MedChemExpress	Cat# HY-B0468
SC79	MedChemExpress	Cat# HY-18749
LY294002	MedChemExpress	Cat# HY-10108
Wheat germ agglutinin-FITC	Sigma-Aldrich	Cat# L4895
MitoSOX™ Red	Beyotime	Cat# S0061
MitoTracker Green	Beyotime	Cat# C1048
LysoTracker red	Beyotime	Cat# C1046

**Critical commercial assays**		

NT-proBNP ELISA Kit	Immunoway	Cat# KE1750
DHE staining kit	Servicebio	Cat# G1746
JC-1 Mitochondrial Membrane Potential Assay Kit	Beyotime	Cat# C2006
M5 Total RNA Extraction Reagent	Mei5 Biotechnology	Cat# MF034
M5 Super Plus qPCR RT Kit	Mei5 Biotechnology	Cat# MF166
M5 HiPer SYBR Premix EsTaq	Mei5 Biotechnology	Cat# MF787

**Deposited data**		

RNA-seq data	This paper	GEO: GSE305470
Proteomics data	This paper	iProX: IPX0013041000

**Experimental models: Cell lines**		

H9c2 rat cardiomyoblasts	Haixing Biosciences	TCR-C607
RAW264.7 murine macrophages	Haixing Biosciences	TCM-C766

**Experimental models: Organisms/strains**		

Mouse: C57BL/6 wild type male	Experimental Animal Center of Shanxi Medical University	N/A

**Oligonucleotides**		

si-Thbs1-1/2/3 sequences	Sangon Biotech	See Supplemental Document S1, Table S1
qRT-PCR primers	Sangon Biotech	See Supplemental Document S1, Table S2

**Recombinant DNA**		

pcDNA3.1(+)-Thbs1-Flag plasmid	Sangon Biotech	N/A

**Software and algorithms**		

ImageJ	NIH	version1.52; https://imagej.nih.gov/ij/
GraphPad Prism 10	GraphPad	https://www.graphpad.com/scientific-software/prism/; version 10
VevoStrain Analysis Software	FUJIFILM VisualSonics	N/A
Image Lab	Bio-Rad	N/A
HISAT2	N/A	2.0.5
featureCounts	N/A	1.5.0-p3
DESeq2	N/A	1.20.0
clusterProfiler	N/A	3.8.1
GSEA	N/A	v3.0

**Other**		

Vevo 2100 Imaging System	FUJIFILM VisualSonics	N/A
CODA Tail-cuff BP System	Kent Scientific	N/A
JEM-1400FLASH TEM	JEOL (Japan)	N/A

### Experimental model and study participant details

#### Experimental models

##### Animals

Healthy male C57BL/6 mice (6–8 weeks old) were obtained from the Experimental Animal Center of Shanxi Medical University (Taiyuan, China). All animal procedures were approved by the Ethics Committee of the First Hospital of Shanxi Medical University (Approval No. DWYJ-2024-014) and were conducted in accordance with institutional and national guidelines for the care and use of laboratory animals. Mice were housed under standard laboratory conditions (25 ± 1°C, 12-h light/dark cycle) with *ad libitum* access to food and water.

#### HFpEF model establishment

A well-characterized “two-hit” HFpEF model was generated as previously described.^48^ Male C57BL/6 mice were fed a high-fat diet (HFD; 60% kcal from fat, D12492, Research Diets) and received L-NAME (0.5 g/L, Sigma-Aldrich) in drinking water (pH 7.4) for 12 consecutive weeks. Control mice were fed standard chow and given regular drinking water. After 12 weeks, cardiac function, body weight, and blood pressure were assessed.

Only male mice were used in this study to avoid confounding effects of sex hormones on cardiac remodeling and metabolic phenotype. Therefore, potential sex differences in HFpEF pathophysiology were not assessed in this study.

#### Cell lines

Two established cell lines were used:H9c2 rat cardiomyoblasts and RAW264.7 murine macrophages (both purchased from Haixing Biosciences, Suzhou, China).

Cells were cultured in DMEM (PYG0073, Boster, Wuhan, China) supplemented with 10% fetal bovine serum and 1% penicillin–streptomycin under standard conditions (37°C, 5% CO_2_). All cell lines were routinely tested and confirmed to be mycoplasma-free.

To simulate an HFpEF-like inflammatory microenvironment,^49^^,^^50^^,^^51^^,^^52^ RAW264.7 macrophages were stimulated with 200 ng/mL ultrapure lipopolysaccharide (LPS; L4391, Sigma-Aldrich, St. Louis, MO, USA) for 6 h to provide a priming signal, followed by 2 mM adenosine triphosphate (ATP; A6559, Sigma-Aldrich) for 30 min to activate the NLRP3 inflammasome and generate macrophage-conditioned medium (MCM). This two-step stimulation protocol has been widely used in recent HFpEF studies to reproduce inflammasome-related cytokine secretion and macrophage-driven paracrine injury in cardiomyocytes. Subsequently, H9c2 cardiomyocytes were treated with MCM combined with isoproterenol (ISO; HY-B0468, MedChemExpress, Monmouth Junction, NJ, USA) for 24 h to mimic the combined inflammatory and adrenergic stress observed in HFpEF.

Pharmacologic modulation of the PI3K/Akt/mTOR pathway was achieved by pretreating H9c2 cells with 10 μM LY294002 (PI3K inhibitor; HY-10108, MedChemExpress) or 10 μM SC79 (an Akt activator; HY-18749, MedChemExpress) for 1 h before stimulation.

For gene manipulation, *Thbs1* expression was modulated by siRNA-mediated knockdown and plasmid-based overexpression approaches. Three siRNAs targeting *Thbs1* (si-*Thbs1*-1/2/3; Sangon Biotech, Shanghai, China) and a pcDNA3.1(+)-*Thbs1*-Flag expression plasmid (Sangon Biotech, Shanghai, China) were used for transient transfection with RNATransMate reagent or Lipofectamine 3000 (Sangon Biotech, Shanghai, China), respectively. Transfection efficiency was evaluated 48 h post-transfection by RT-qPCR and Western blotting. The sequences of siRNAs are provided in Supplementary Document S1, Table S1.

### Method details

#### AAV vector construction and *in vivo* delivery

To achieve cardiomyocyte-specific knockdown of *Thbs1 in vivo*, an adeno-associated virus serotype 9 vector (AAV9-cTnT-sh*Thbs1*) was constructed. The vector expresses a short hairpin RNA (shRNA) targeting mouse *Thbs1* (target sequence: 5′-GGA GAA GAC TTA GAC AAT A-3′) embedded within a miR-30 backbone, transcriptionally driven by the cardiac troponin T (cTnT) promoter (Hanbio Biotechnology, Shanghai, China). Embedding the shRNA in the miR-30 scaffold enhances its processing efficiency through the endogenous microRNA biogenesis pathway and reduces potential off-target effects or cytotoxicity commonly observed with conventional U6 promoter–driven shRNA systems. The cTnT promoter restricts shRNA expression to cardiomyocytes, ensuring cardiac-specific *Thbs1* silencing without affecting non-cardiac tissues.

A non-targeting scramble shRNA control vector (AAV9-cTnT-shScramble, hereafter referred to as AAV9-NC) was used as the negative control. For clarity, AAV9-NC refers to a scramble shRNA control rather than an empty vector. To validate the cardiomyocyte specificity of the cTnT promoter, a parallel AAV9-cTnT-EGFP reporter vector was generated and systemically delivered. Four weeks after tail-vein injection, hearts were harvested, cryosectioned, and subjected to immunofluorescence staining, which confirmed EGFP expression in ventricular cardiomyocytes.

Male C57BL/6 mice were randomized to receive 100 μL of AAV9-cTnT-sh*Thbs1* or AAV9-NC via tail-vein injection at 1.8 × 10^12^ vg/mL under brief isoflurane anesthesia (induction 5%, maintenance 1.5–2%). AAV9 administration was performed at the onset of HFpEF induction. Based on prior studies, AAV9-mediated knockdown typically reaches maximal efficiency 3–4 weeks post-injection and maintains sustained suppression throughout the 12-week modeling period, coinciding with the completion of HFpEF induction. A subset of animals was euthanized 4 weeks post-injection to verify knockdown efficiency and tissue specificity by qRT-PCR and Western blotting of left ventricular tissue. The remaining mice proceeded through the experimental protocol and were euthanized at the study endpoint for comprehensive tissue collection and analysis.

#### Conventional echocardiography and Doppler imaging

Transthoracic echocardiography was performed using a Vevo 2100 imaging system (FUJIFILM VisualSonics, Canada). Mice were anesthetized with 1.5% isoflurane in 98.5% oxygen and placed on a temperature-controlled platform. Systolic function was assessed using M-mode imaging at the mid-ventricular level in conscious mice, while diastolic function was evaluated under anesthesia using pulsed-wave and tissue Doppler imaging, as previously described.^53^ All echocardiographic measurements were performed by investigators blinded to treatment allocation. Measurements were repeated at least three times, and averaged values were reported.

#### Speckle-tracking echocardiography and strain analysis

Global longitudinal strain (GLS) was assessed from B-mode images (parasternal long-axis view) using VevoStrain software. Myocardial velocity and displacement were calculated in both long- and short-axis planes. Negative strain values reflect longitudinal myocardial shortening. Six ventricular segments were analyzed, and average peak GLS values were calculated. The detailed value of echocardiography results is in Supplementary Document S1, Tables S2 and S3.

#### Blood pressure measurement

Systolic blood pressure was measured non-invasively using the tail-cuff method (CODA System, Kent Scientific). Mice were placed in restrainers on a 37°C platform. After acclimatization, measurements were taken for four consecutive days, with a minimum of eight readings per session. Mean systolic blood pressure was calculated from all valid measurements.

#### Measurement of cardiac marker enzymes

Serum N-terminal pro-B-type natriuretic peptide (NT-proBNP) levels were measured using a commercial ELISA kit (KE1750, Immunoway, USA) according to the manufacturer’s protocol.

#### Histopathological analysis

Heart tissues were fixed in 4% paraformaldehyde, paraffin-embedded, and sectioned (5 μm thickness). Hematoxylin-eosin (HE) and Masson’s trichrome staining were performed using commercial kits (Servicebio, Wuhan, China). All histological assessments and quantifications were performed by investigators blinded to treatment allocation. Collagen content was quantified using ImageJ software.

Wheat germ agglutinin (WGA) staining was performed on paraffin-embedded sections after fixation. Sections were incubated with WGA-FITC (1:100, Sigma-Aldrich) for 1 h at 37°C. Nuclei were counterstained with DAPI. Cardiomyocyte cross-sectional area was analyzed using ImageJ.

Immunohistochemistry was performed using anti-Thbs1 primary antibody (1:5000, ab267388, Abcam) and HRP-conjugated secondary antibody. Signal was visualized using chromogenic substrate and observed under a light microscope.

#### Transmission electron microscopy (TEM)

Heart tissues were fixed in 3% glutaraldehyde and post-fixed with 1% osmium tetroxide. After dehydration and embedding in Epon 812 resin, ultrathin sections were stained with uranyl acetate and lead citrate. Mitochondrial ultrastructure was visualized using a JEM-1400FLASH transmission electron microscope (JEOL, Japan).

#### Oxidative stress detection

Reactive oxygen species (ROS) production was assessed in heart tissue cryosections using DHE staining (G1746, Servicebio) and in cultured cells using MitoSOX Red (S0061, Beyotime, Shanghai, China). Sections or cells were incubated with fluorescent probes (5 μM) at 37°C for 30 min in the dark and imaged using a fluorescence microscope. ImageJ was used for quantitative analysis.

#### Mitochondrial function assays

Mitochondrial membrane potential (MMP) was assessed using the JC-1 assay kit (C2006, Beyotime). Cells were incubated with JC-1 working solution at 37°C for 20 min, washed, and imaged under a fluorescence microscope. Red-to-green fluorescence ratio was used as an indicator of MMP.

#### MitoTracker and LysoTracker colocalization assay

H9c2 cells were seeded in 12-well plates and subjected to experimental treatments. To visualize mitochondrial–lysosomal interactions, cells were incubated with 200 nM MitoTracker Green (Beyotime, Cat# C1048) and 50 nM LysoTracker red (Beyotime, Cat# C1046) for 30 min at 37°C in the dark. Fluorescence images were directly captured using a fluorescence microscope. The images are intended for qualitative visualization of mitochondria–lysosome colocalization.

#### Immunofluorescence staining

Deparaffinized heart sections or fixed cells were subjected to antigen retrieval, blocked with 3% BSA, and incubated overnight at 4°C with primary antibodies: Anti-LC3 (Santa Cruz Biotechnology, sc-271625, 1:300) and Anti-VDAC1 (Servicebio, GB111939, 1:300). The sections were then incubated with corresponding secondary antibodies, including Cy3-conjugated Goat Anti-Mouse IgG (H + L) (Servicebio, GB21301, 1:300) and Alexa Fluor 488-conjugated Goat Anti-Rabbit IgG (H + L) (Servicebio, GB25303, 1:400). Finally, nuclei were counterstained with DAPI. Fluorescence signals were visualized using a fluorescence microscope.

#### Quantitative reverse-transcription PCR (RT-qPCR)

Total RNA was extracted using M5 Total RNA Extraction Reagent (MF034, Mei5, Beijing, China). Reverse transcription was performed using the M5 Super Plus qPCR RT Kit (MF166, Mei5), and qPCR was carried out using M5 HiPer SYBR Premix EsTaq (MF787, Mei5) on a CFX96 system (C1000 Touch Thermal Cycler, Bio-Rad, CA, USA). GAPDH served as an internal control, and the 2ˆ-ΔΔCt method was used for relative quantification. Primer sequences are listed in Supplementary Document S1, Table S4.

#### Co-immunoprecipitation (Co-IP)

Co-immunoprecipitation assays were performed to examine the interaction between Thbs1 and integrin β1 (ITGB1) in H9c2 cells. Briefly, cells were lysed in IP lysis buffer supplemented with protease and phosphatase inhibitors. Cell lysates were incubated with 2 μg of rabbit anti-Thbs1 antibody (Abcam, Cat# ab267388) or mouse anti-integrin β1 (ITGB1, A-4) antibody (Santa Cruz Biotechnology, Cat# sc-374429) overnight at 4°C with gentle rotation. Protein A/G agarose beads were then added and incubated for 2 h at 4°C. Immunoprecipitates were washed three times with lysis buffer, eluted in SDS sample buffer, and subjected to Western blot analysis. For detection, corresponding HRP-conjugated secondary antibodies were used: anti-rabbit IgG and anti-mouse IgG. Signals were visualized using enhanced chemiluminescence (ECL).

#### Western blotting

Total proteins were extracted using RIPA buffer with protease inhibitors, separated by SDS-PAGE, and transferred to PVDF membranes. Membranes were blocked with 5% milk, incubated overnight at 4°C with the following primary antibodies diluted in blocking buffer: anti-ANP (Boster, A01318-1, 1:1000), anti-BNP (Abmart, PS00154, 1:1000), anti-Thbs1 (Abcam, ab267388, 1:1000), anti-LC3B (Abways, CY5992, 1:1000), anti-p62 (Abways, CY5546, 1:2000), anti-PINK1 (Cell Signaling Technology, #6946, 1:1000), anti-Parkin (Abways, CY6641, 1:1000), anti-p-PI3K (Abways, CY6427, 1:1000), anti-PI3K (Abways, CY6915, 1:1000), anti-*p*-Akt (Abways, CY6569, 1:1000), anti-Akt (Abways, CY5561, 1:1000), anti-*p*-mTOR (Abways, CY6571, 1:1000), anti-mTOR (Abways, CY5306, 1:1000), anti-β-Tubulin (Abways, AB0039, 1:10000) and anti-GAPDH (Abways, AB0037, 1:5000). After washing, membranes were incubated with HRP-conjugated secondary antibodies (Abcam, ab6721,1:10000) diluted in blocking buffer 1 h at room temperature. Bands were visualized using the ChemiDoc Imaging System (Bio-Rad), and intensities quantified with Image Lab software.

#### Transcriptomic and proteomic analyses

Left ventricular (LV) myocardium was dissected from control and HFpEF mice for multi-omics analysis. Each group included three independent biological replicates (*n* = 3), with tissues collected from distinct mice. For transcriptomic profiling, total RNA was extracted, and RNA quality assessment, library construction, and sequencing were performed by Novogene Bioinformatics Co., Ltd. (Beijing, China). High-throughput sequencing was conducted on the Illumina NovaSeq platform. Clean reads were aligned to the mouse reference genome using HISAT2, and gene expression levels were quantified with featureCounts. Differential gene expression analysis was performed using the DESeq2 package. Genes with an adjusted *p*-value <0.05 and an absolute log_2_ fold change ≥2 were defined as differentially expressed genes (DEGs). KEGG pathway enrichment analysis was conducted using the clusterProfiler R package. RNA-seq data are available at the NCBI Gene Expression Omnibus (GEO) database with accession number GSE305470.

For proteomic analysis, total protein was extracted from the same LV samples and subjected to tandem mass tag (TMT)-based quantitative proteomic profiling at Novogene. Protein samples were digested with trypsin, labeled with TMT reagents, and analyzed by high-resolution liquid chromatography–tandem mass spectrometry (LC-MS/MS). Differentially expressed proteins (DEPs) were identified using a *p*-value <0.05 and fold change >1.2 or <0.83. Functional enrichment of DEPs was performed using clusterProfiler, and gene set enrichment analysis (GSEA) was applied to identify significantly enriched pathways at the proteomic level. Proteomics data have been deposited in integrated proteome resources (iProX) under accession number IPX0013041000.

To examine the correlation between transcriptomic and proteomic alterations, integrated analysis was conducted by matching DEGs and DEPs based on gene symbols. Overlapping molecules with consistent expression trends at both mRNA and protein levels were identified. Additionally, joint analysis of transcriptomic and proteomic datasets was performed to reveal common differentially expressed targets and shared signaling pathways involved in the pathogenesis of HFpEF.

### Quantification and statistical analysis

Data are presented as mean ± standard deviation (SD), with the number of biological replicates (n, independent animals or independent cell culture experiments) indicated in each figure legend. Technical replicates were averaged prior to analysis where appropriate. Statistical analyses were performed using GraphPad Prism version 10 (GraphPad Software, USA). Comparisons between two groups were performed using unpaired two-tailed Student’s *t* test for normally distributed data with equal variances. For comparisons among three or more groups, one-way ANOVA followed by Tukey’s post hoc test was applied. Nonparametric tests (Mann–Whitney U test for two groups; Kruskal–Wallis test followed by Dunn’s post hoc test for multiple groups) were used when data were not normally distributed or variances were unequal. Normality and variance homogeneity were assessed based on sample characteristics.

Animals were randomly assigned to experimental groups, and investigators were blinded to group allocation during echocardiographic, histological, and biochemical analyses. Sample sizes were determined based on pilot experiments and prior studies to ensure adequate statistical power. No data points were excluded. Statistical significance was defined as ∗*p* < 0.05, ∗∗*p* < 0.01, ∗∗∗*p* < 0.001, and ∗∗∗∗*p* < 0.0001. The asterisks indicate statistical significance based on the tests used. All statistical details, including *n* values, tests used, and definition of replicates, are provided in figure legends and the results section.

## Supplemental information

Document S1. Figures S1–S9 and Tables S1–S4

Data S1. Original western blotting raw bands
