# Supplementary material for: Thrombospondin 1 aggravates cardiac remodeling in heart failure with preserved ejection fraction by inhibiting mitophagy
Source: iScience. 2026 Jan 7;29(2):114639. doi: 10.1016/j.isci.2026.114639 (PMC12857411; doi:10.1016/j.isci.2026.114639)
Supplement: Document S1. Figures S1–S9 and Tables S1–S4 [file mmc1.pdf]

iScience, Volume *29*

Supplemental information

Thrombospondin 1 aggravates cardiac remodeling in heart failure with preserved ejection fraction by inhibiting mitophagy

Xingpeng Bu, Shuo Sha, Zhenzhen Zhang, Sicheng Bian, Shuhui Feng, Chunxia Li, Lei Wang, and Huanzhen Chen

Supplementary Materials

Figure S1. Activation of the PI3K/Akt/mTOR signaling pathway in the myocardium of HFpEF mice

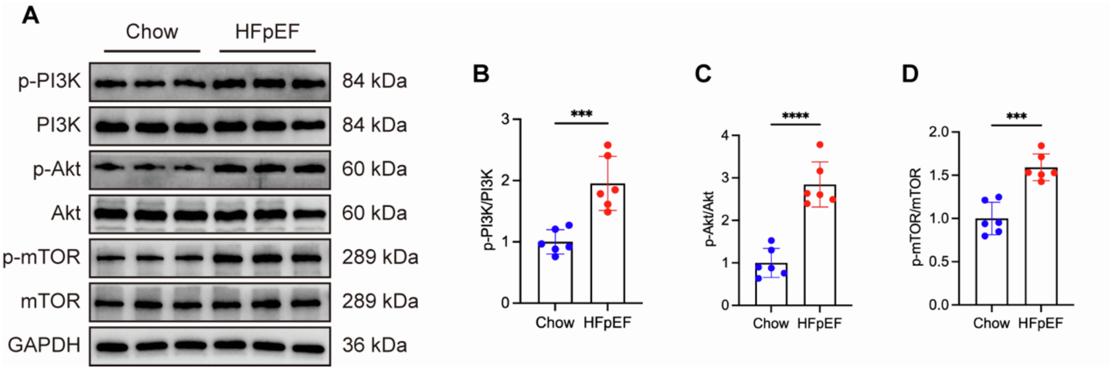

(A) Representative Western blot images showing phosphorylated and total PI3K, Akt, and mTOR in left ventricular tissue from HFpEF and chow-fed control mice. (B–D) Quantification of protein phosphorylation ratios: p-PI3K/PI3K (B), p-Akt/Akt (C), and p-mTOR/mTOR (D) ($n = 6$ biological replicates per group, each representing one mouse). Data are presented as mean ± SD. ***$p < 0.001$, ****$p < 0.0001$; unpaired two-tailed Student's t-test.

Figure S2. Validation of cardiac-specific *Thbs1* knockdown in vivo by AAV9

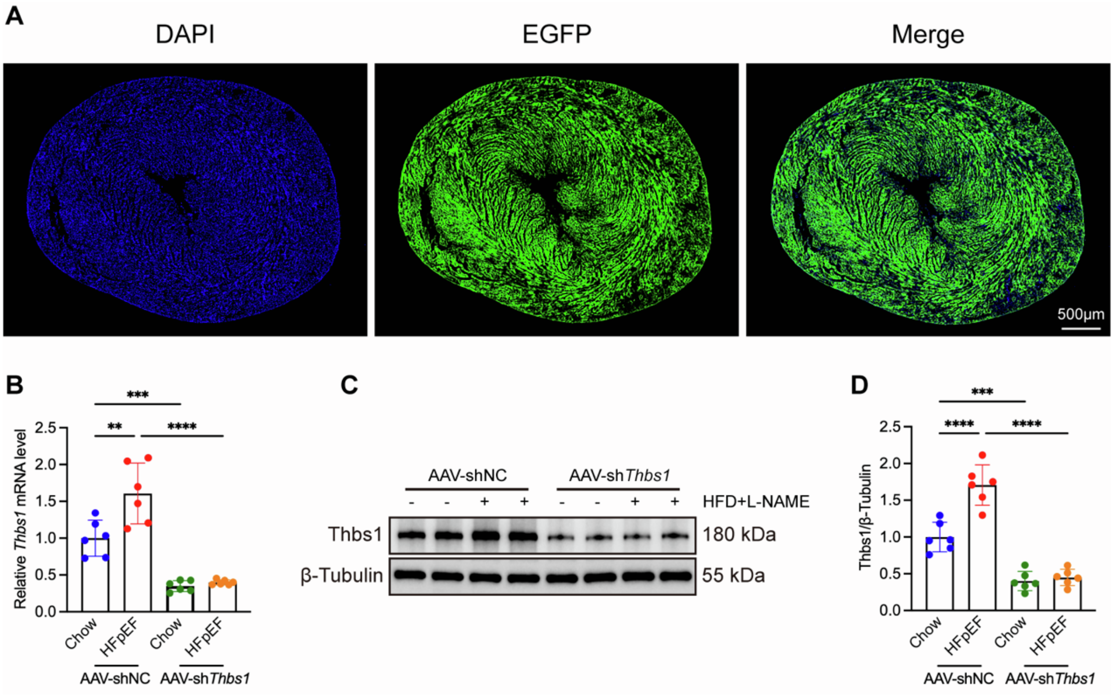

(A) Immunofluorescence staining of cardiac cryosections from mice injected with AAV9-cTnT-EGFP, showing specific EGFP expression restricted to ventricular cardiomyocytes (scale bar, 500µm). (B) RT-qPCR analysis showing reduced *Thbs1* mRNA expression in mouse hearts four weeks after AAV9-sh*Thbs1* injection compared with AAV9-shNC controls (n = 6 biological replicates per group, each representing one mouse). (C) Representative Western blot images showing Thbs1 protein expression. (D) Densitometric quantification of Thbs1 protein levels normalized to β-Tubulin (n = 6 biological replicates per group). Data are presented as mean ± SD. $^{**}p < 0.01$, $^{***}p < 0.001$, $^{****}p < 0.0001$; one-way ANOVA followed by Tukey's post hoc test.

Figure S3. Hemodynamic and structural assessment following cardiac-specific *Thbs1* knockdown in HFpEF mice

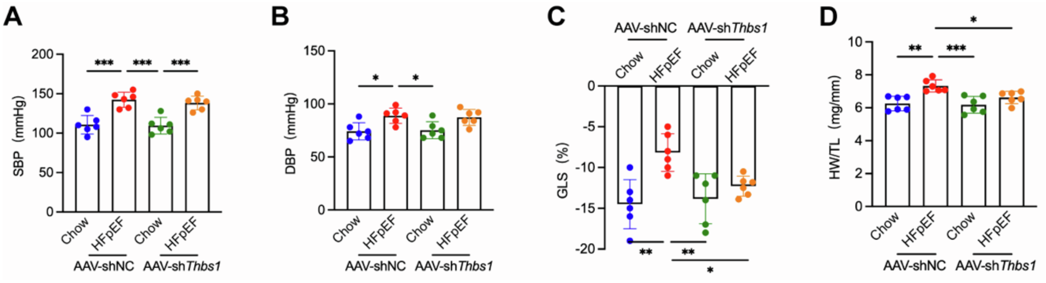

(A) Systolic blood pressure (SBP) and (B) diastolic blood pressure (DBP) in AAV-sh*Thbs1* and AAV-shNC mice subjected to high-fat diet (HFD) plus L-NAME treatment for 12 weeks ($n = 6$ biological replicates per group, each representing one mouse). (C) Global longitudinal strain (GLS) measured by echocardiography ($n = 6$). (D) Heart weight normalized to tibia length (HW/TL) ratio ($n = 6$). Data are presented as mean ± SD. *$p < 0.05$, **$p < 0.01$, ***$p < 0.001$; one-way ANOVA followed by Tukey's post hoc test.

Figure S4. Validation of siRNA-mediated *Thbs1* knockdown efficiency in H9c2 cardiomyocytes

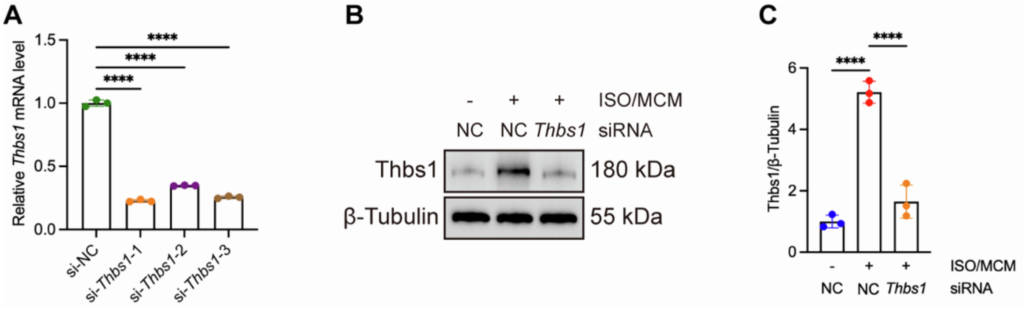

(A) RT-qPCR analysis of *Thbs1* mRNA expression after transfection with three different siRNAs (si-*Thbs1*-1, si-*Thbs1*-2, and si-*Thbs1*-3) compared with negative control (si-NC) (n = 3 biological replicates). (B) Representative Western blot images showing Thbs1 protein expression following siRNA transfection. (C) Densitometric quantification of Thbs1 protein knockdown efficiency based on Western blot analysis (n = 3). Data are presented as mean ± SD. ****$p < 0.0001$; one-way ANOVA followed by Tukey's post hoc test.

Figure S5. *Thbs1* knockdown attenuates cardiomyocyte hypertrophy *in vitro*

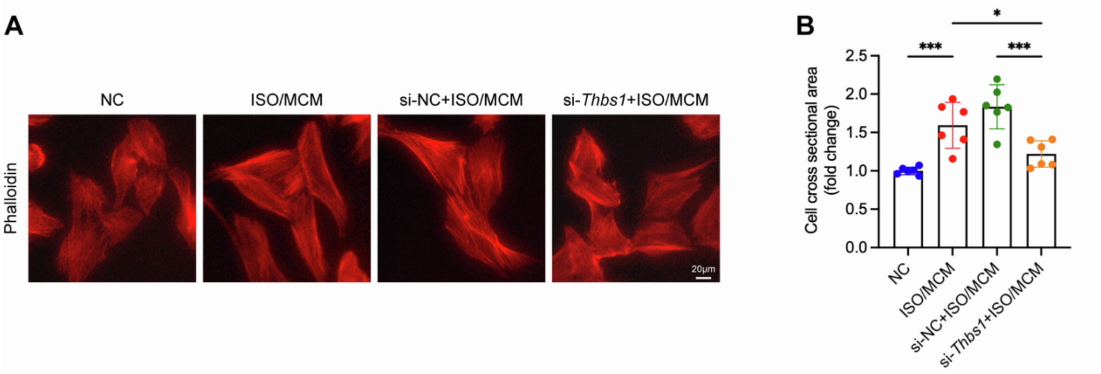

(A) Representative fluorescence images of H9c2 cells stained with phalloidin-TRITC to visualize F-actin and assess cellular hypertrophy under ISO and MCM stimulation, with or without si-*Thbs1* transfection (scale bar, 20μm). (B) Quantification of cardiomyocyte surface area ($n = 6$ biological replicates per group, each measured in triplicate). Data are presented as mean ± SD. *$p < 0.05$, ***$p < 0.001$; one-way ANOVA followed by Tukey's post hoc test.

Figure S6. Validation of Thbs1 overexpression in H9c2 cardiomyocytes

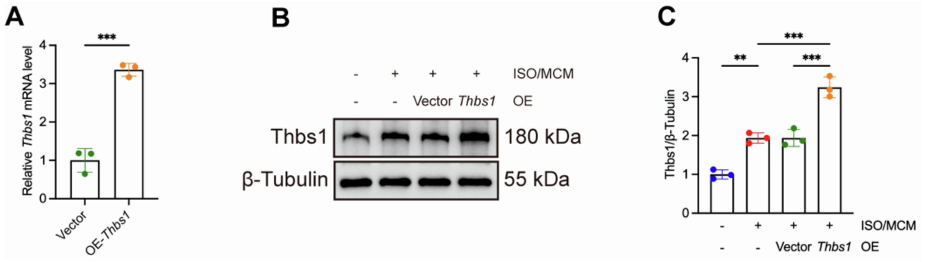

(A) RT-qPCR analysis confirming increased *Thbs1* mRNA expression following pcDNA3.1-Flag-*Thbs1* transfection (n = 3 biological replicates, each measured in triplicate). (B) Representative Western blot images showing elevated Thbs1 protein expression in transfected H9c2 cells. (C) Densitometric quantification of Thbs1 protein levels normalized to β-Tubulin (n = 3 biological replicates, each measured in triplicate). Data are presented as mean ± SD. **$p < 0.01$, ***$p < 0.001$; one-way ANOVA followed by Tukey's post hoc test.

Figure S7. *Thbs1* overexpression exacerbates cardiomyocyte hypertrophy *in vitro*

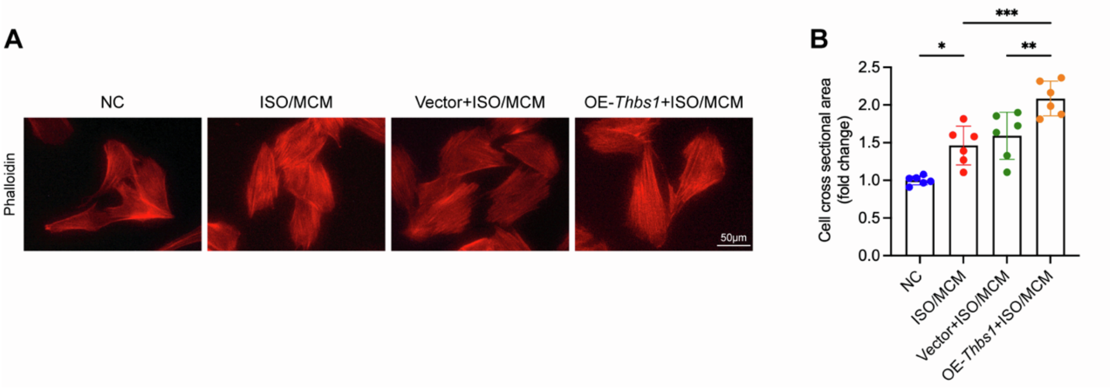

(A) Representative images of H9c2 cells stained with Phalloidin TRITC to visualize F-actin and assess cellular hypertrophy under ISO and MCM stimulation (scale bar, 50μm). (B) Quantification of cardiomyocyte surface area (*n* = 6 biological replicates, each measured in triplicate). Data are presented as mean ± SD. **p* < 0.05, ****p* < 0.001; one-way ANOVA followed by Tukey's post hoc test.

Figure S8. *Thbs1* knockdown suppresses PI3K/Akt/mTOR pathway activation in HFpEF mouse hearts

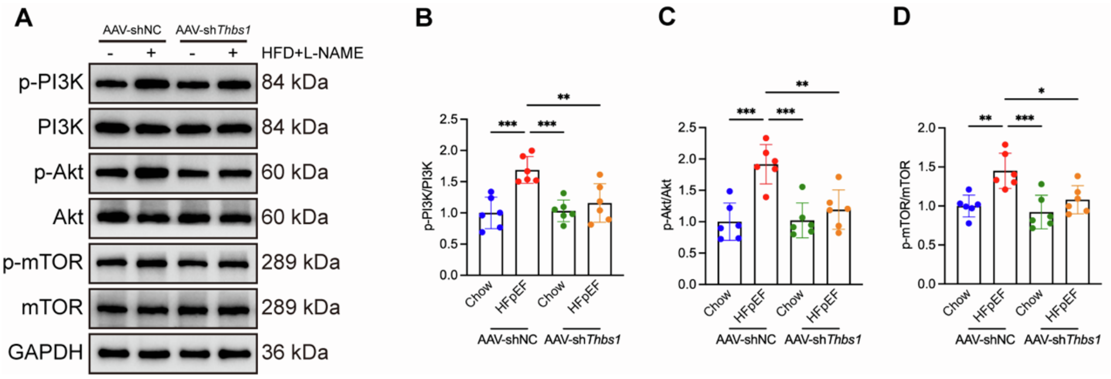

(A) Representative Western blot images showing phosphorylated and total PI3K, Akt, and mTOR in left ventricular myocardial tissue from each experimental group. (B–D) Quantification of p-PI3K/PI3K (B), p-Akt/Akt (C), and p-mTOR/mTOR (D) ratios (n = 6 biological replicates per group, each measured in duplicate). Data are presented as mean ± SD. *$p < 0.05$, **$p < 0.01$, ***$p < 0.001$; one-way ANOVA followed by Tukey's post hoc test.

Figure S9. PI3K/Akt/mTOR inhibition reverses hypertrophic effects of *Thbs1* overexpression in H9c2 cells

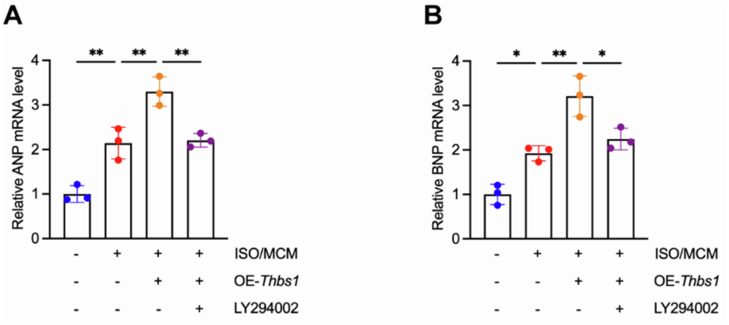

(A and B) Relative mRNA expression of hypertrophic markers ANP (A) and BNP (B) assessed by RT-qPCR in H9c2 cells under four experimental conditions ($n = 3$ biological replicates per group, each measured in triplicate). Data are presented as mean ± SD. *$p < 0.05$, **$p < 0.01$; one-way ANOVA followed by Tukey's post hoc test for multiple-group comparisons.

Table S1. The siRNA sequences of *Thbs1*

NAME	Forward (5' to 3' sequence)	Reverse (5' to 3' sequence)
si-*Thbs1*-1	CGCUGUUUGUCCAAGAAGA	UCUUCUUGGACAAACAGCG
si-*Thbs1*-2	GGGAGAUGUCAAUGACAAU	AUUGUCAUUGACAUCUCCC
si-*Thbs1*-3	GCUCCAGUUCAGCUACCAA	UUGGUAGCUGAACUGGAGC

Table S2. Echocardiography analysis of cardiac function in Chow and HFpEF mice

	Chow	HFpEF	*p* value
LVID, d (mm)	3.90 ± 0.14	4.06 ± 0.16	0.0981
LVPW, d (mm)	0.79 ± 0.09	0.94 ± 0.15	0.0611
LVEF (%)	62.67 ± 4.72	61.5 ± 5.75	0.7090
LVFS (%)	33.17 ± 3.31	32.5 ± 2.59	0.7058
E/A	1.22 ± 0.15	2.21 ± 0.50	0.0010
E/e'	24.85 ± 3.70	35.98 ± 9.10	0.0211
GLS (%)	-13.67 ± 3.20	-8.5 ± 3.08	0.0174

Data are presented as mean ± SD. n = 6 biological replicates per group (each value represents one mouse; each averaged from three technical measurements). Statistical significance was determined using unpaired two-tailed Student’s t-test.

Table S3. Echocardiography analysis of cardiac function in AAV-shNC and AAV-sh*Thbs1* mice with or without HFD/L-NAME treatment

	AAV-shNC Chow	AAV-shNC HFpEF	AAV-sh*Thbs1* Chow	AAV-sh*Thbs1* HFpEF
LVID, d (mm)	3.87 ± 0.13	4.05 ± 0.19	3.99 ± 0.22	3.92 ± 0.09
LVPW, d (mm)	0.79 ± 0.08	0.93 ± 0.14	0.80 ± 0.08	0.87 ± 0.10
LVEF (%)	63.67 ± 4.72	61.67 ± 5.47	63.33 ± 3.33	62.33 ± 2.16
LVFS (%)	33.5 ± 3.27	31.83 ± 2.64	32.83 ± 2.48	32.5 ± 1.05
E/A	1.23 ± 0.16	2.16 ± 0.67	1.20 ± 0.16	1.53 ± 0.21
E/e'	24.62 ± 3.61	35.49 ± 7.64	25.22 ± 3.33	27.44 ± 3.54
GLS (%)	-14.50 ± 3.02	-8.17 ± 2.31	-15.17 ± 3.76	-12.28 ± 1.22

Data are presented as mean ± SD. n = 6 biological replicates per group (each value represents one mouse; each averaged from three technical measurements). Statistical significance was determined by one-way ANOVA followed by Tukey's post hoc test.

Table S4. The primers for RT-qPCR in this study

**Gene**	**Forward (5' to 3' sequence)**	**Reverse (5' to 3' sequence)**
Thbs1	GTGAGGTTTGTCTTTGGAACCA	GTTGTTGTCAAGGGTAAGAAGGA
IL-1β	GCAACTGTTCCTGAACTCAACT	ATCTTTTGGGGTCCGTCAACT
IL-6	TAGTCCTTCCTACCCCAATTTCC	TTGGTCCTTAGCCACTCCTTC
TNF-α	TCTCATCAGTTCTATGGCCC	GGGAGTAGACAAGGTACAAC
ANP	GGGGAAGTCAACCCGTCTCA	TCAATCCTACCCCCGAAGCA
BNP	TTCCGGATCCAGGAGAGACTT	CCTAAAACAACCTCAGCCCGT
GAPDH	TGACCTCAACTACATGGTCTACA	CTTCCCATTCTCGGCCTTG
