# Supplementary material for: Thrombospondin 1 aggravates cardiac remodeling in heart failure with preserved ejection fraction by inhibiting mitophagy
Source: iScience. 2026 Jan 7;29(2):114639. doi: 10.1016/j.isci.2026.114639 (PMC12857411; doi:10.1016/j.isci.2026.114639)
Supplement: Data S1. Original western blotting raw bands [file mmc2.pdf]

Data S1. Original western blotting raw bands

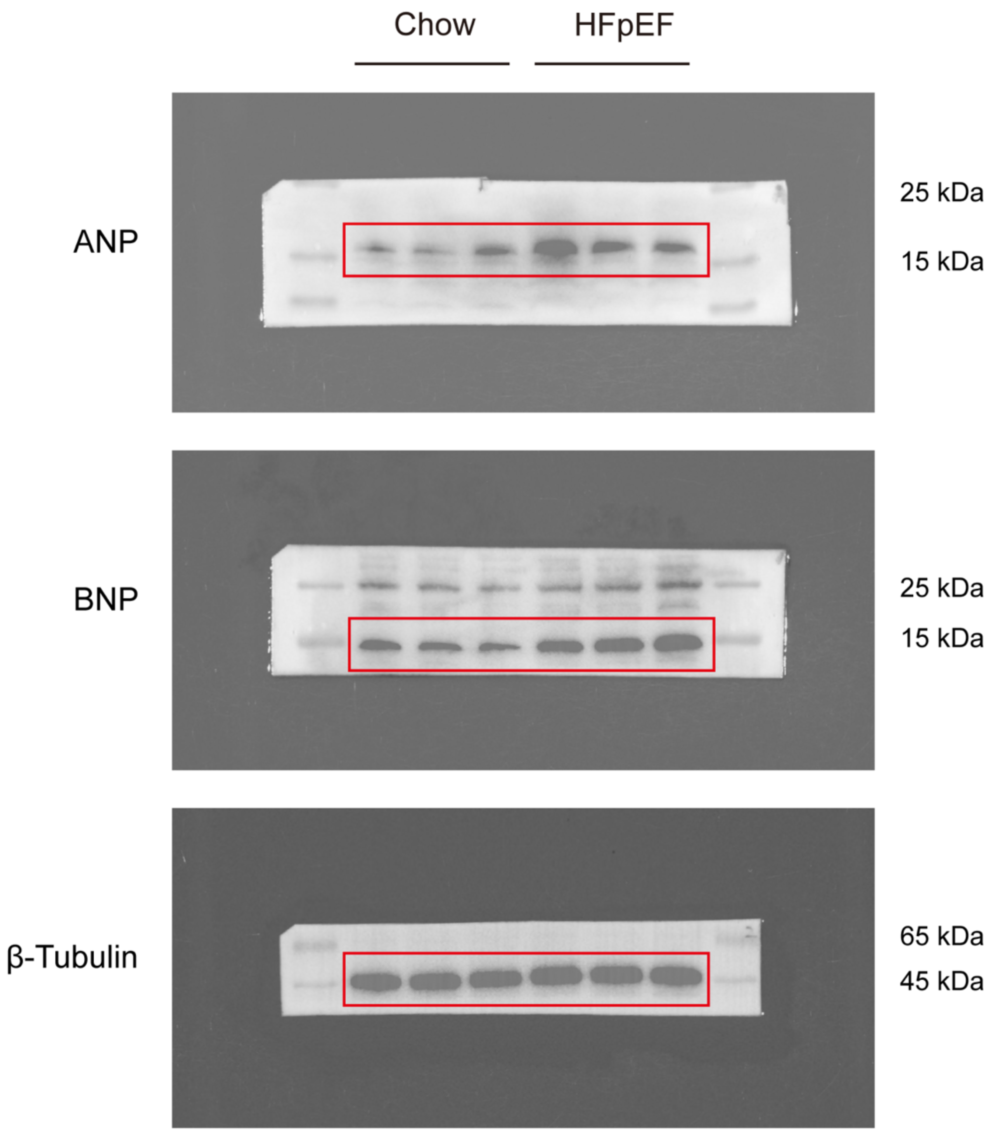

Figure.1P Western Blotting Raw Bands

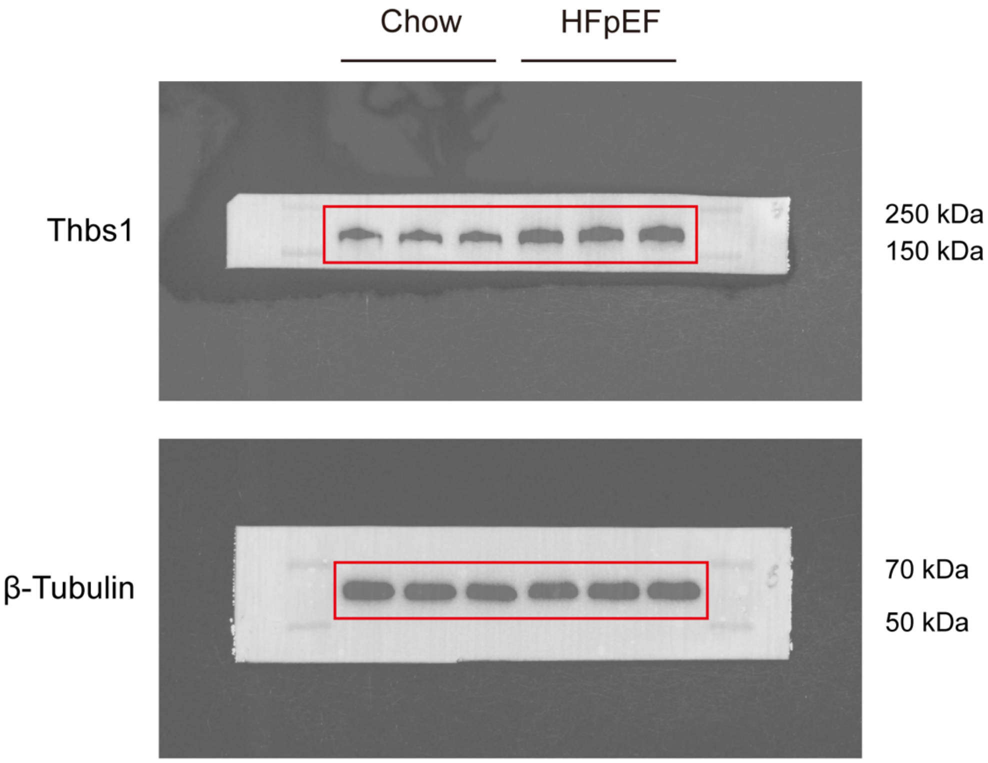

Figure.2J Western Blotting Raw Bands

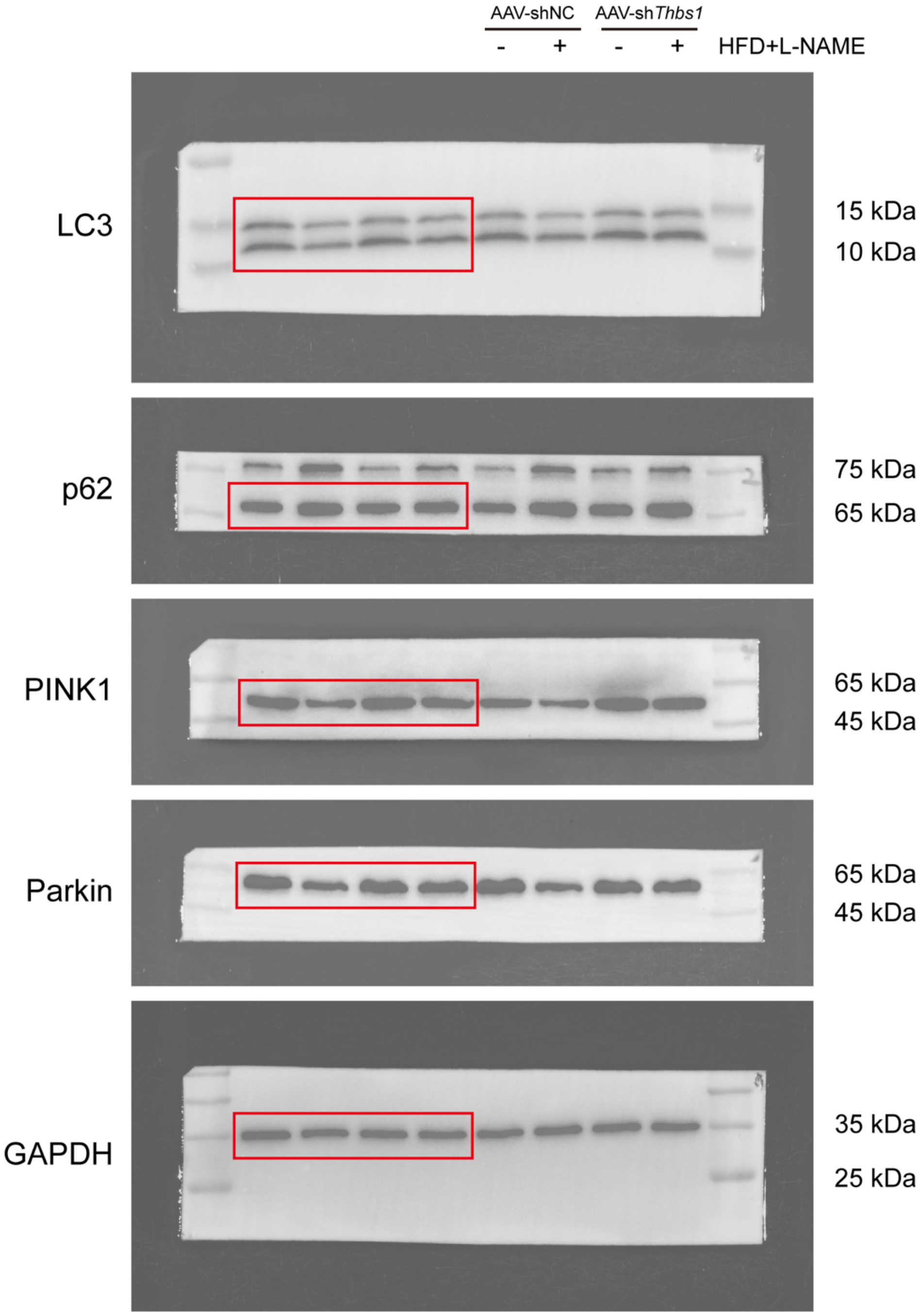

Figure.4C Western Blotting Raw Bands

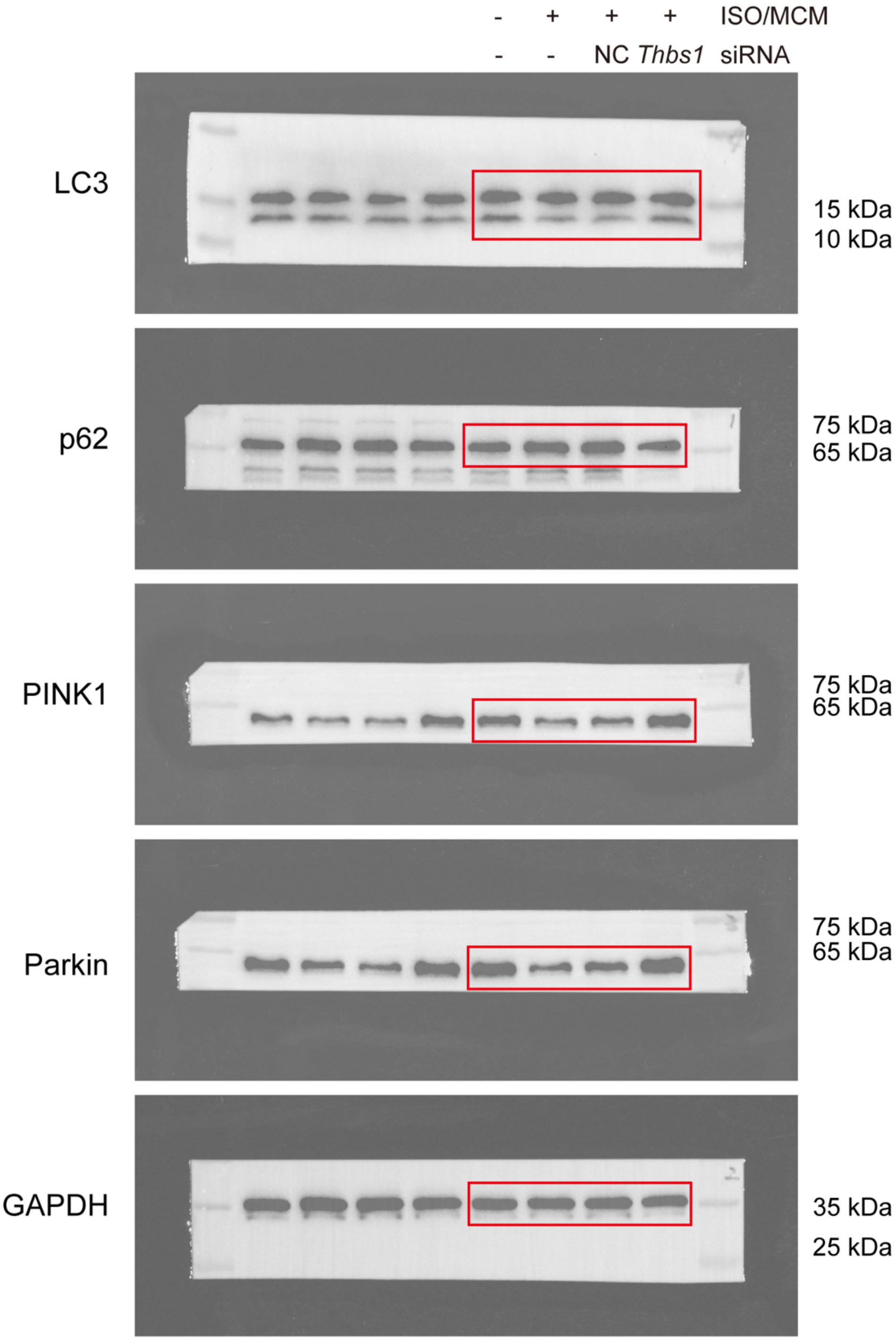

Figure.5I Western Blotting Raw Bands

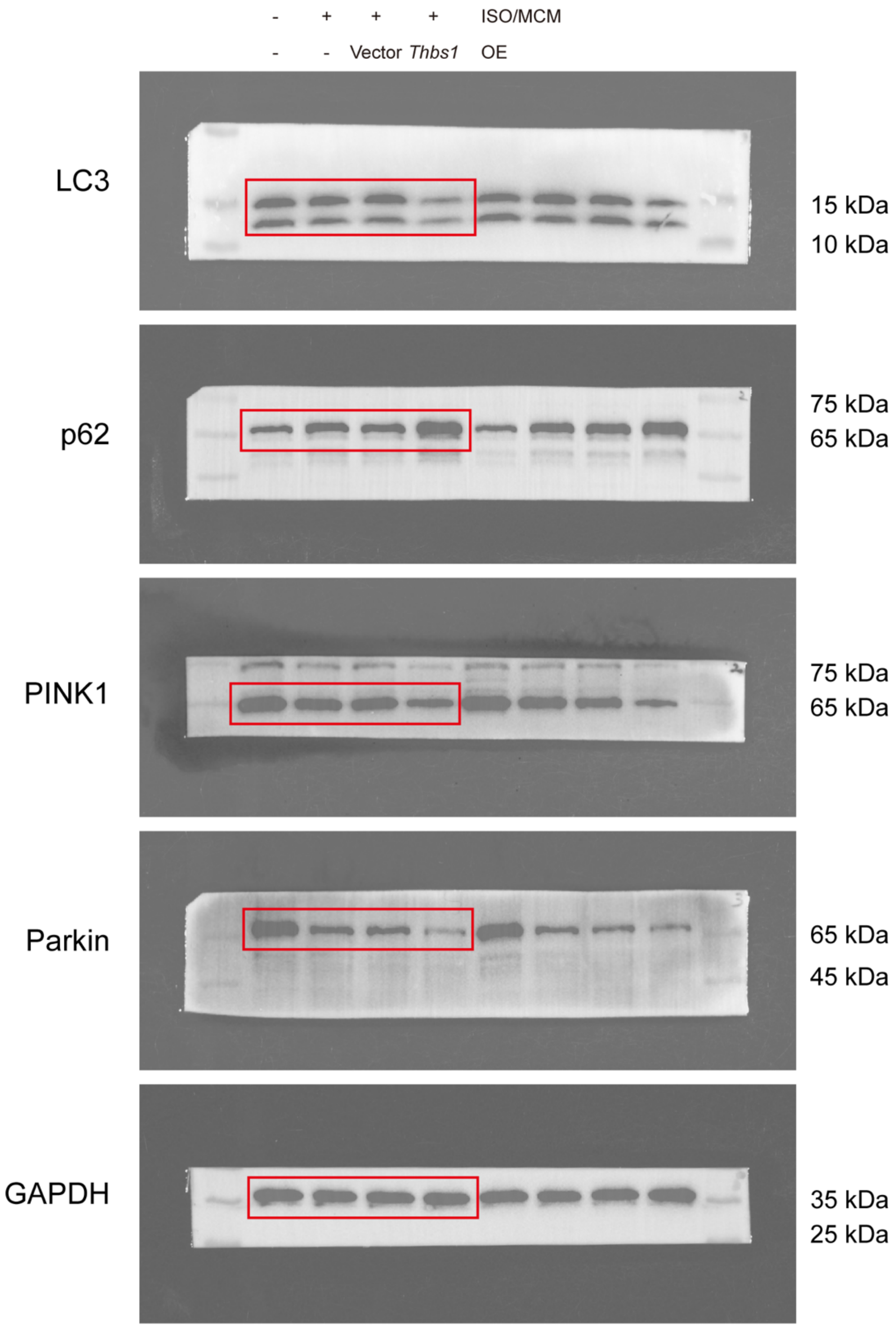

Figure.6H Western Blotting Raw Bands

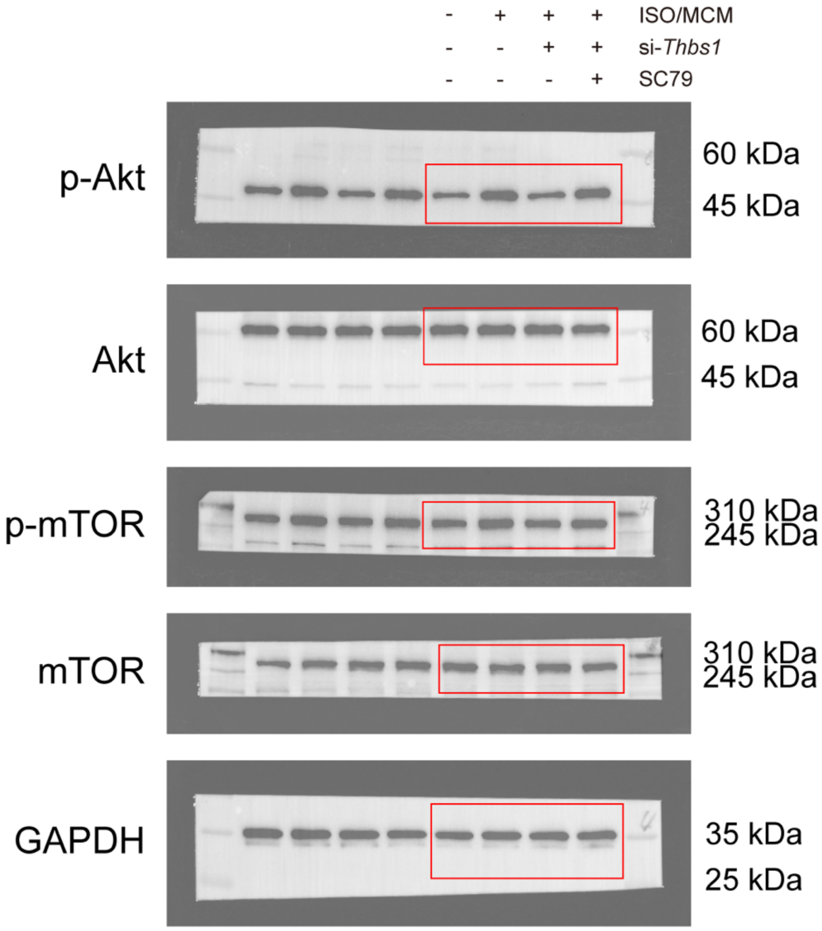

Figure.7A Western Blotting Raw Bands

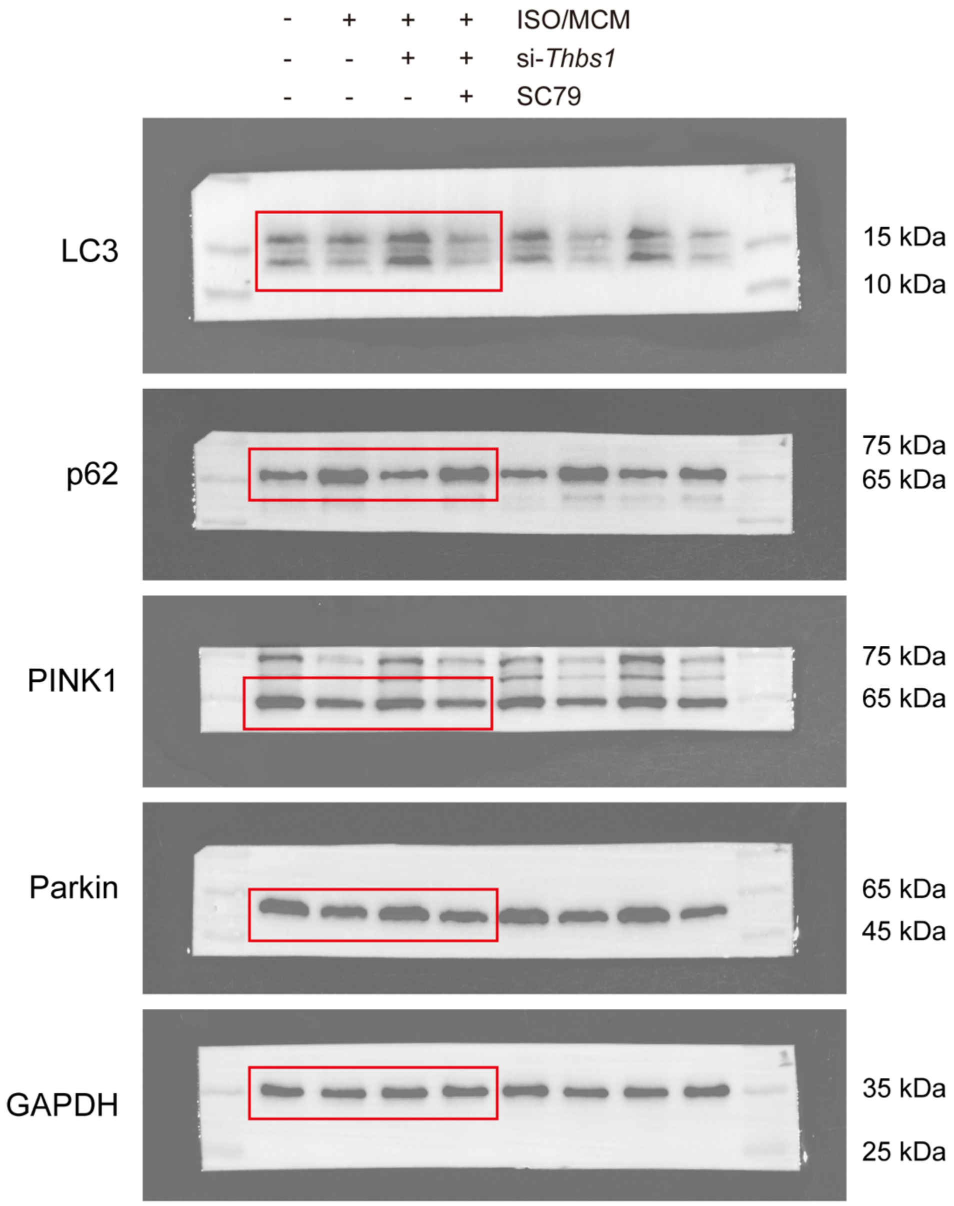

Figure.7I Western Blotting Raw Bands

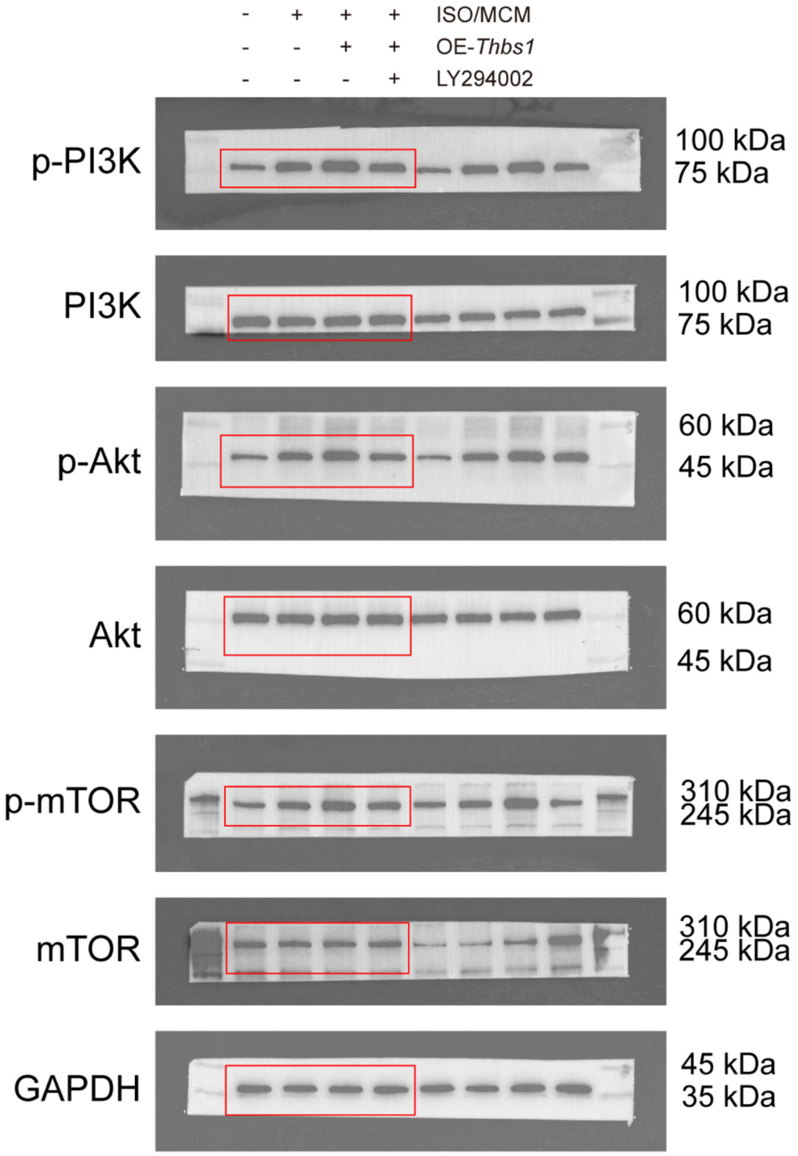

Figure.8A Western Blotting Raw Bands

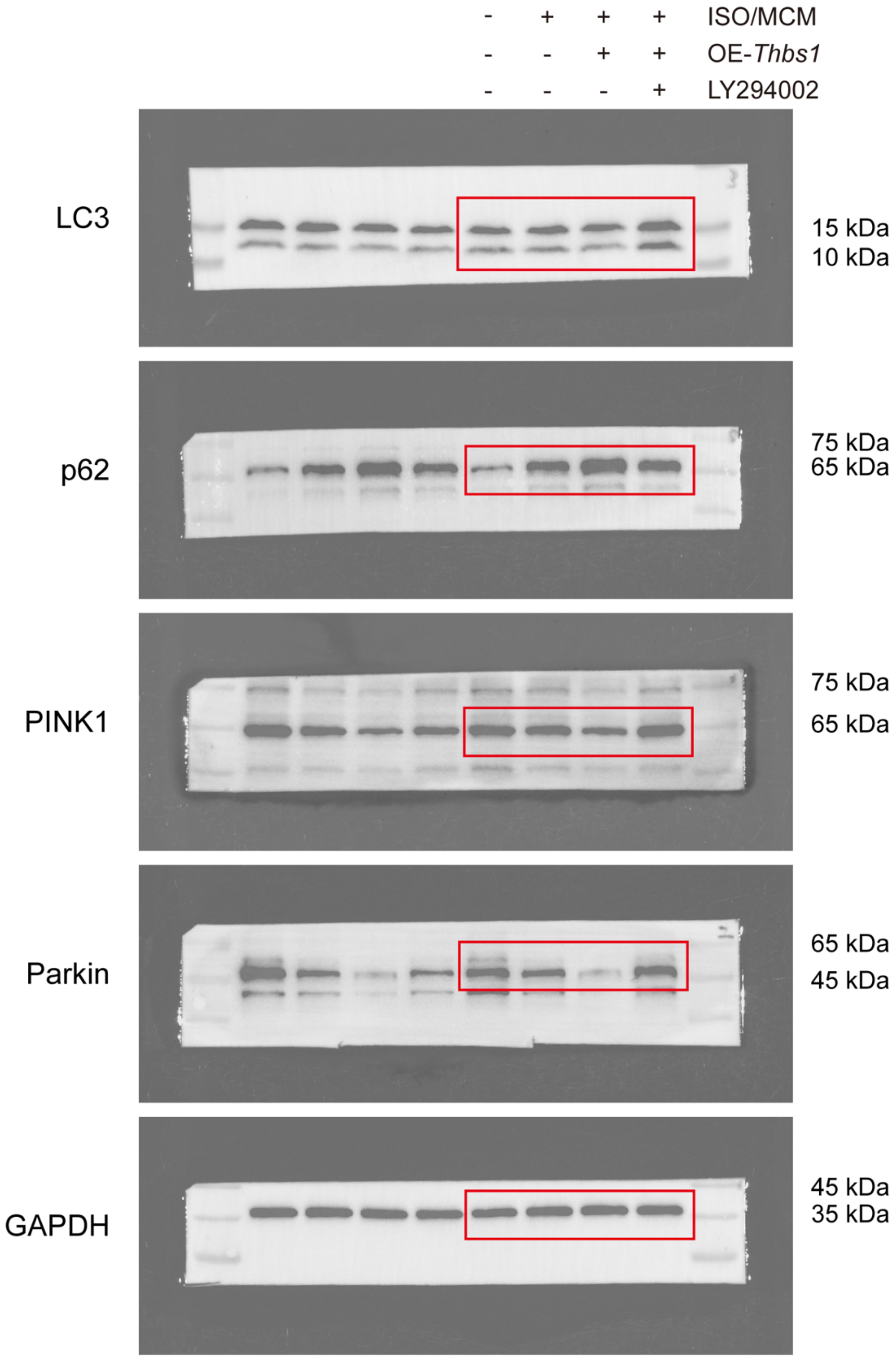

Figure.8H Western Blotting Raw Bands

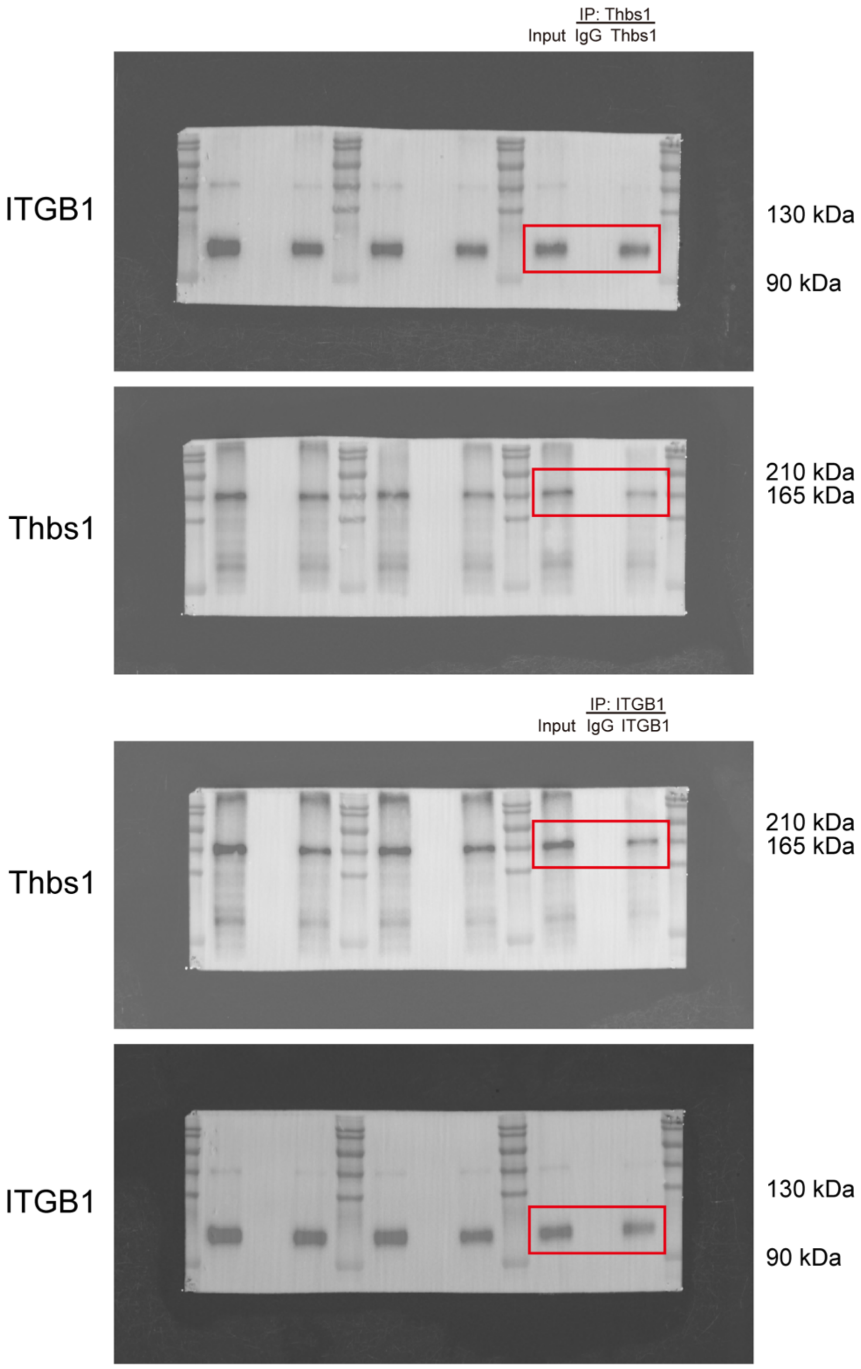

Figure.8M Western Blotting Raw Bands

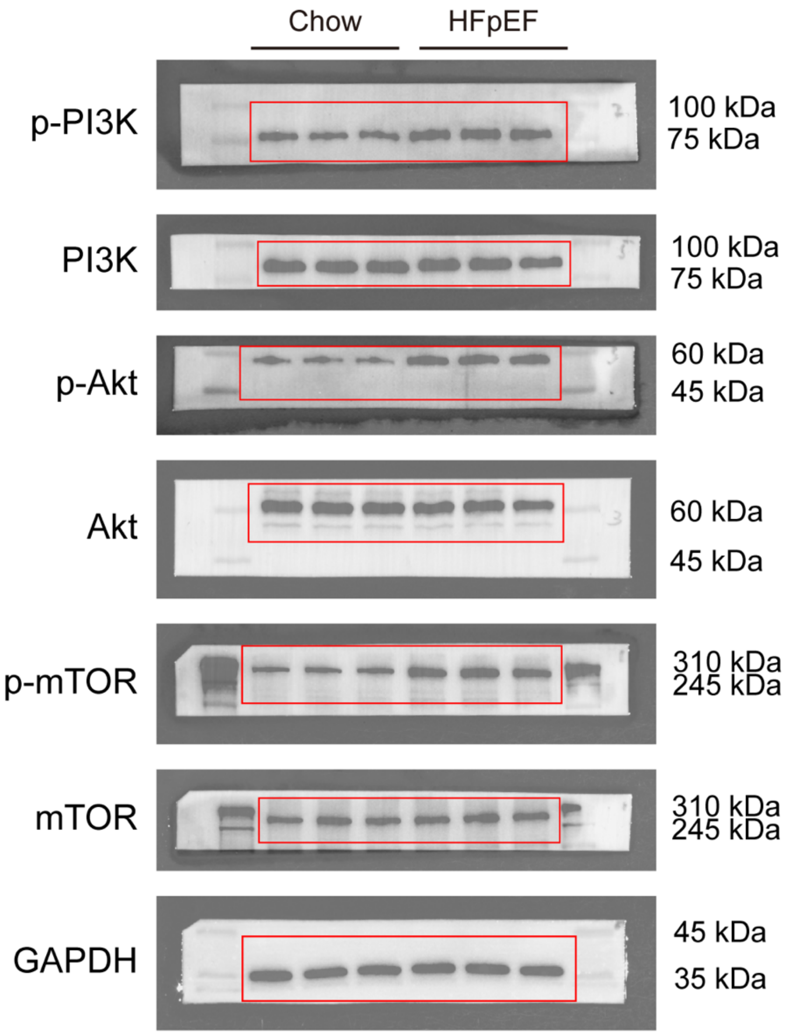

Figure.S1A Western Blotting Raw Bands

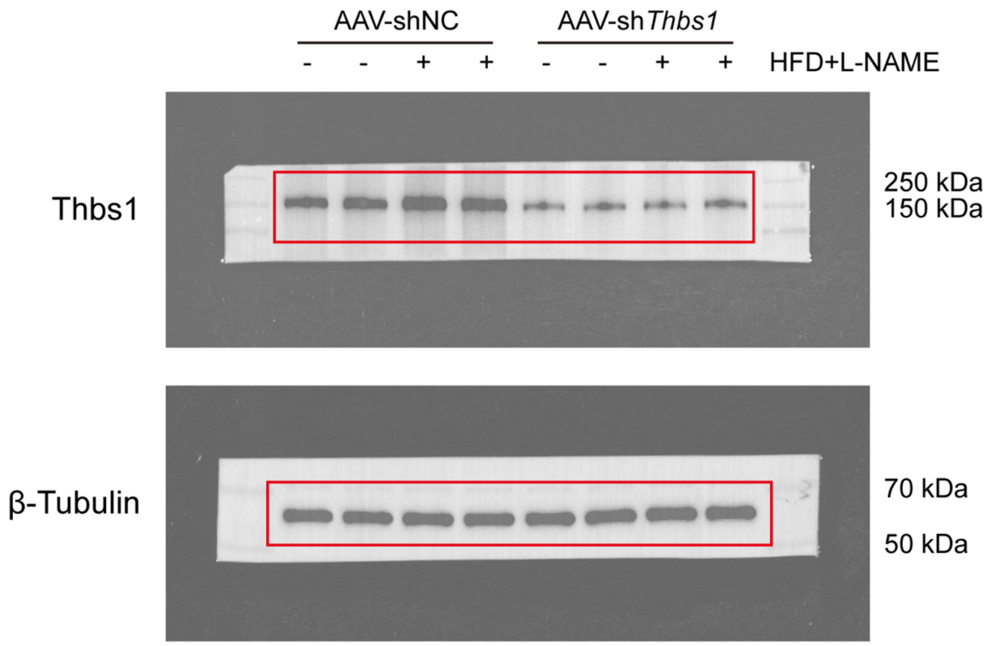

Figure.S2C Western Blotting Raw Bands

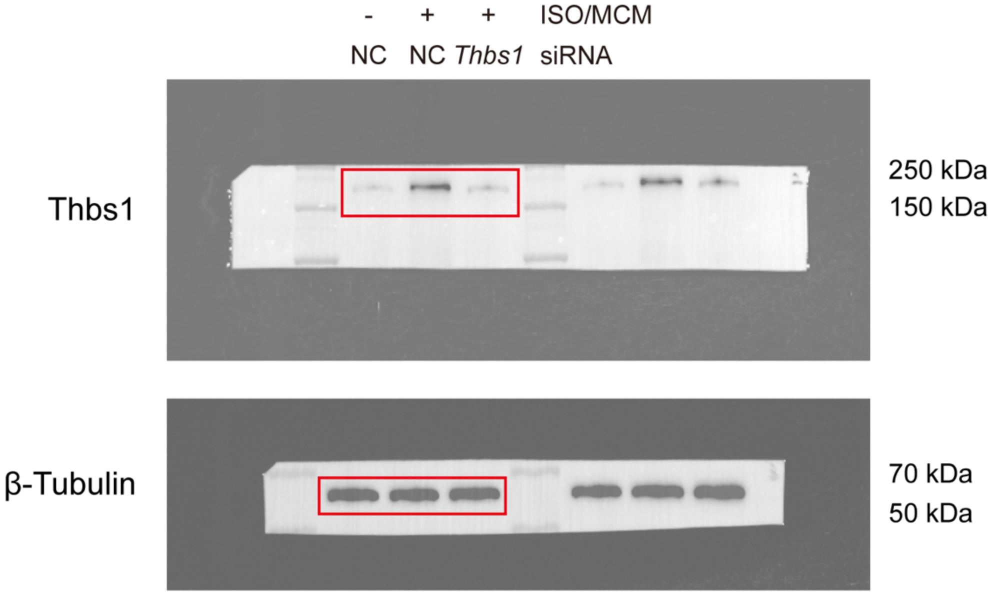

Figure.S4B Western Blotting Raw Bands

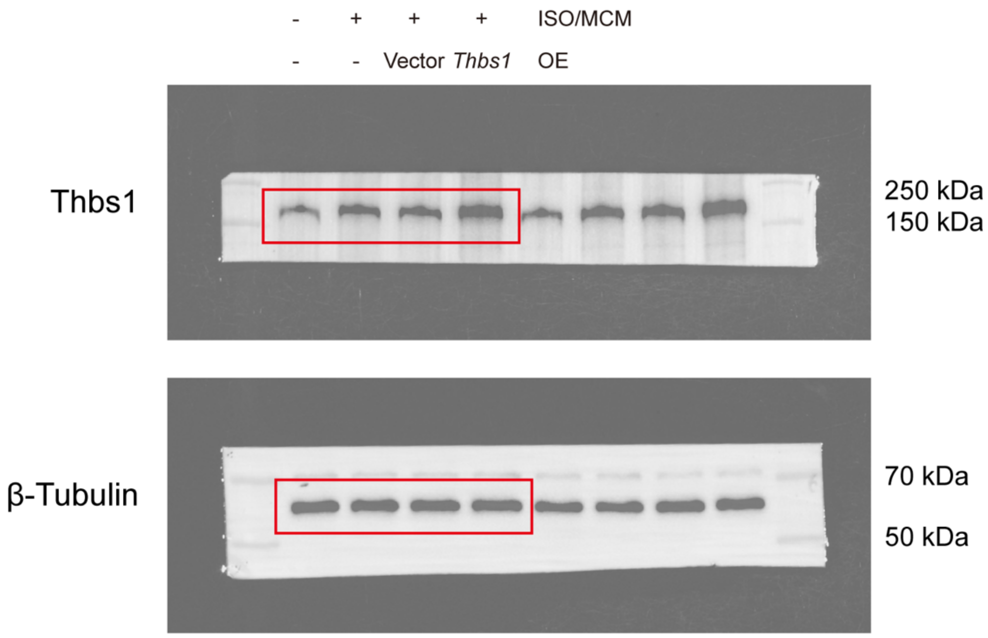

Figure.S6B Western Blotting Raw Bands

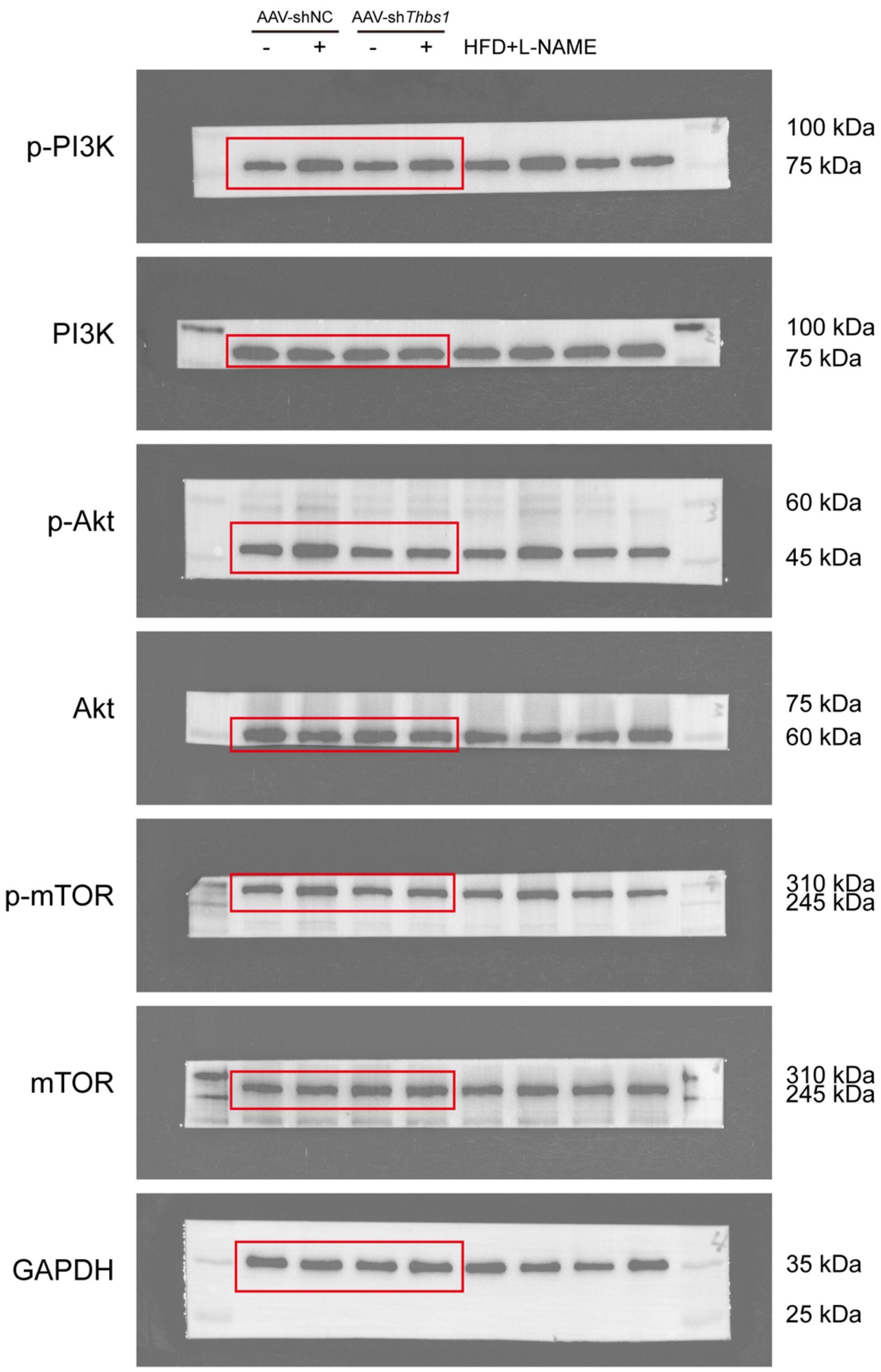

Figure.S8A Western Blotting Raw Bands
